# Rosetta:MSF:NN: Boosting performance of multi-state computational protein design with a neural network

**DOI:** 10.1371/journal.pone.0256691

**Published:** 2021-08-26

**Authors:** Julian Nazet, Elmar Lang, Rainer Merkl

**Affiliations:** Institute of Biophysics and Physical Biochemistry, University of Regensburg, Regensburg, Germany; University of Michigan, UNITED STATES

## Abstract

Rational protein design aims at the targeted modification of existing proteins. To reach this goal, software suites like Rosetta propose sequences to introduce the desired properties. Challenging design problems necessitate the representation of a protein by means of a structural ensemble. Thus, Rosetta multi-state design (MSD) protocols have been developed wherein each state represents one protein conformation. Computational demands of MSD protocols are high, because for each of the candidate sequences a costly three-dimensional (3D) model has to be created and assessed for all states. Each of these scores contributes one data point to a complex, design-specific energy landscape. As neural networks (NN) proved well-suited to learn such solution spaces, we integrated one into the framework Rosetta:MSF instead of the so far used genetic algorithm with the aim to reduce computational costs. As its predecessor, Rosetta:MSF:NN administers a set of candidate sequences and their scores and scans sequence space iteratively. During each iteration, the union of all candidate sequences and their Rosetta scores are used to re-train NNs that possess a design-specific architecture. The enormous speed of the NNs allows an extensive assessment of alternative sequences, which are ranked on the scores predicted by the NN. Costly 3D models are computed only for a small fraction of best-scoring sequences; these and the corresponding 3D-based scores replace half of the candidate sequences during each iteration. The analysis of two sets of candidate sequences generated for a specific design problem by means of a genetic algorithm confirmed that the NN predicted 3D-based scores quite well; the Pearson correlation coefficient was at least 0.95. Applying Rosetta:MSF:NN:enzdes to a benchmark consisting of 16 ligand-binding problems showed that this protocol converges ten-times faster than the genetic algorithm and finds sequences with comparable scores.

## Introduction

Computational protein design has become an important tool in molecular biology [1]. Different approaches and protocols have proven their reliability for a broad range of applications. To name just a few design problems, for a protein under study, the informed user can increase thermostability [2, 3], alter the binding of ligands [4, 5], redesign interactions with other proteins [6, 7] or design novel catalytic sites [8]. Moreover, the *de novo* design of catalytically active proteins is feasible [9, 10] as well as antibody redesign [11, 12].

For challenging design problems that require the modelling of structural flexibility, the traditional, single-state design (SSD) strategy that is optimal for finding a sequence for one structurally fixed backbone is not sufficient. For example, enzymes adopt different conformations during a catalytic cycle and more generally, all important biological effects are best represented by an ensemble of conformational states; for a review see [13]. This is why multi-state design (MSD) protocols have been introduced, which score a single sequence with respect to conformationally different backbones modelled as states [14–24]. Moreover, MSD allows for negative design, i.e., the computation of sequences that destabilize certain states related to misfolded conformations or an undesired binding interaction [25]. However, the more precise MSD approach has its price due to the demands for higher computational efforts needed for the identification of appropriate sequences: Considering *m* states requires scoring each candidate sequence *m* times and combining these scores to a global “fitness” value in order to identify sequences that are optimal for all states. MSD approaches have demonstrated their superiority in applications like the prediction of mutational tolerance in enzymes [26], the understanding of thermal adaptation of enzymes [27], the design of influenza antibodies [28], multi-specific interfaces [29], and multi-substrate enzymes [30].

A well-proven and highly flexible software suite supporting highly diverse problems of protein design is Rosetta [31] and several Rosetta-based MSD protocols have been implemented [14, 15, 28]. To determine the fitness of a sequence during the search phase, the 3D residue positions, whose occupancy can be varied by the software protocol, are decorated with the considered amino acid side chains. A key element of this assessment is to find an optimal combination of side chain orientations [32]. Building these optimized 3D (3D_opt_) models consumes most of the computational time needed for the whole protein design protocol. If the occupancy of *n* residue positions is unconstrained for a design task, optimal rotamer combinations have to be found and assessed for 20^*n*^ different 3D_opt_ models in an SSD and for *m*×20^*n*^ 3D_opt_ models in an MSD experiment. Thus, even for design problems of moderate complexity, a hybrid method that does not require the computation of a costly 3D_opt_ model to score each of the candidate sequences might drastically reduce computation time of Rosetta’s protein design protocols.

This search for optimal sequences can be considered a problem of multi-dimensional regression, were every combination of amino acid residues yields one data point of the design-specific energy landscape. Due to the superior runtime, the usage of a simple regression model seems an attractive means for the scoring of these residue combinations. However, structure and function of proteins often depend on nonlinear and nonadditive relationships between the physical properties of residues [33]. Especially for protein engineering and design, it is highly recommended to consider these complex interactions [34], which strongly argues against the usage of simple regression models. In contrast, neural networks (NNs) have proven well-suited to solve complex classification and regression problems of computational biology [35, 36]. Thus, we explored, whether we can utilize an NN in a hybrid approach to rapidly sample candidate sequences during Rosetta protocols. More specifically, we wanted to test whether the existence of a moderate number of 3D_opt_ models is sufficient to teach an NN the energy landscape of a specific design problem. Thus, we implemented the multi-state framework ROSETTA:MSF:NN and used benchmark datasets to confirm that NNs can learn the design-specific energy landscapes of protein design. We found that ROSETTA:MSF:NN converges 10-times faster than our previous protocol and samples alternative areas of sequence space.

## Materials and methods

### Datasets used for the initial performance test

The dataset HisB_GA_raw_ consisted of 48,588 tuples $des\_seq_{j}^{raw}=(aa_{1}^{j},\ldots ,aa_{14}^{j},RS_{3\mathrm{DM}}^{j})$ that were generated by means of Rosetta:MSF:GA:enzdes during 500 iterations of a genetic algorithm (GA) for an ongoing design project. Based on one-hot encoding, each 20-dimensional vector $aa_{pos}^{j}$ represented one amino acid residue at position *pos* of a candidate sequence *j*. This experiment was aimed at the redesign of the 14 residues constituting the ligand-binding site of the bifunctional enzyme HisB-N from *Escherichia coli*. This enzyme hydrolyses l-histidinol phosphate to l-histidinol and phosphate as well as O-phospho-l-serine to l-serine and phosphate. For this MSD approach, 11 states were used that represented slightly different conformations generated by means of a short (1 ns) molecular dynamic simulation seeded with the structure of the N-terminal domain of *E*. *coli* HisB (chain A of PDB-ID 2fpu). The sequences enumerated by the GA are highly similar, due to the preferential introduction of single point mutations.

In order to create a second, non-redundant dataset HisB_GA_nr_ consisting of tuples $des\_seq_{j}^{nr}=(aa_{1}^{j},\ldots ,aa_{14}^{j},RS_{3\mathrm{DM}}^{j})$, the maximal pairwise sequence identity was limited to 70%, which gave rise to 533 sequences. Selecting the sequences randomly, a training dataset consisting of 67% and a test dataset consisting of 33% of the sequences were created both for HisB_GA_raw_ and HisB_GA_nr_.

### Benchmark dataset MD_EnzBench

The dataset MD_EnzBench has been generated previously for benchmarking ligand binding design based on Rosetta:MSF [3]. It has been deduced from molecular dynamics (MD) simulations of length 10 ns generated with YASARA (version 14.7.17) and the YAMBER3 force field that has been parameterized to produce crystal-structure-like protein coordinates [37]. MD_EnzBench consists of 16 proteins *prot*_*k*_ with bound ligand taken from the scientific sequence recovery benchmark of Rosetta [38] and each *prot*_*k*_ is indicated by the PDB-ID. To introduce conformational flexibility during the MD simulations, the ligand has been removed and for each of the 16 apoproteins, 1000 conformations have been sampled at an interval of 10 ps. As a structural basis for the subsequent MSD protocol, protein conformations have been saved every 1 ns and used as states. After sampling, the native ligands have been re-introduced in all conformations of the respective apoproteins by means of PyMOL:superpose [39]. The design and repack shells of all enzymes have been listed previously [2]. All design shell residues have been replaced with alanine and prior to design, all conformations have been energy-minimized by means of Rosetta:fastrelax with backbone constraints.

### Design and implementation of the NN

**Fig 1:**
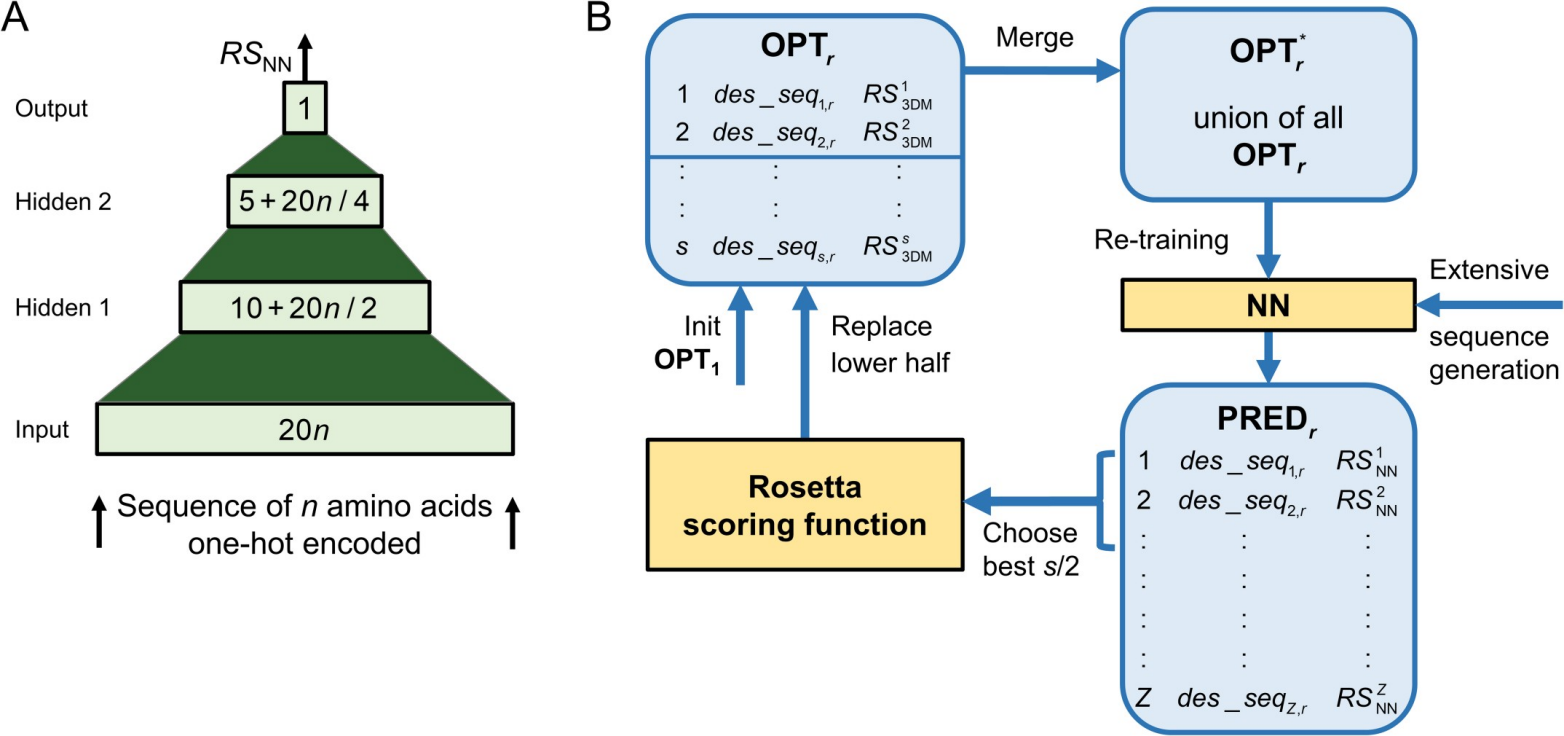
NN architecture and data generation within Rosetta:MSF:NN. (A) The input layer of the NN contains 20*n* neurons that process the 20-dimensional vectors representing the amino acids $aa_{pos}^{j}$ of the *pos* = 1,…,*n* design shell residues under study. The output layer consists of one neuron that determines the *RS*_NN_ value. The first hidden layer consists of 10+20*n*/2 neurons and the second hidden layer of 5+20*n*/4 neurons. All neurons of adjacent layers are fully connected. (B) During all iterations *r* of sequence optimization, Rosetta:MSF:NN administers a pool OPT_*r*_ of *s* sequences, whose *RS*_3DM_ values are known. The union $OPT_{r}^{*}$ of these sequences is growing iteration-wise and used to re-train the NN that has an architecture as in (A). The re-trained NN is utilized for an extensive sequence scan that computes per default the *RS*_NN_ values for *Z* = 2,000,000 randomly generated sequences based on a seed. For *s*/2 sequences possessing highest *RS*_NN_ values, the (costly) *RS*_3DM_ is computed. These sequences and their *RS*_3DM_ values constitute the lower half of the updated set OPT_*r*+1_. For the first iteration, OPT_1_ is initialized with *s* datasets.

The 4-layered architecture of the NN is shown in **Fig 1A**. The input layer consists of 20*n* neurons that are supplied with a one-hot encoding of amino acid residues $aa_{pos}^{j}$ of the *n* design shell residues whose composition constituted the candidate sequence under study. The first hidden layer consists of 10+20*n*/2 neurons and the second hidden layer of 5+20*n*/4 neurons. The output layer consists of one neuron that computes the score *RS*_NN_ as a real value. Each layer is fully connected with the previous layer and no bias is used in any layer.

The NN was created using the python package *Keras* version 2.2.4 and *TensorFlow* 1.12.0 as backend. For initialization, a RandomNormal kernel was used in all layers. Both hidden layers utilize a tanh and the output layer a linear activation function. The network was optimized by means of a stochastic gradient descent with momentum. Prior to training, the scores *RS*_3DM_ computed by Rosetta for 3D models, were converted to z-scores. The model was trained for 100 epochs in incremental mode. Note that we used a lightweight NN that does not require the usage of GPUs.

### Design and data flow of Rosetta:MSF:NN

Our previously introduced approach Rosetta:MSF:GA utilizes the same strategy as a recently published generic program [14]: In an outer loop, a GA is used to search sequence space and for each state, rotamers are optimized in an inner loop. However, Rosetta:MSF:GA does not operate on a population of 100, but on 239 sequences.

In order to allow for a fair comparison with Rosetta:MSF:GA, the novel NN-based approach Rosetta:MSF:NN administers for each iteration *r* a set OPT_*r*_ of *s* = 239 sequences, whose $RS_{3\mathrm{DM}}^{j}$ values are used for their ranking; see **Fig 1B**. These *des*_*seq*_*j*_ sequences represent the amino acid residues $aa_{pos}^{j}$ chosen for the positions *pos* = 1,…,*n* of the design shell under study and for each *des*_*seq*_*j*_, the $RS_{3\mathrm{DM}}^{j}$ value was computed by means of the chosen Rosetta scoring function. Initially, Rosetta:MSF:NN generates the set OPT_1_ consisting of the given seed sequences and mutants, each with a randomly introduced single point mutation [15]. During each iteration *r*, OPT_*r*_ is added to ${\mathrm{OPT}}_{r}^{*}$, which contains the shuffled union of all so far chosen *des*_*seq*_*j*_ data. The updated set ${\mathrm{OPT}}_{r}^{*}$ is used to re-train the NN, which is then utilized to assess an extensive set of *Z* novel sequences. The seeds for these novel sequences are the iteration-specific top-scoring sequences. Per default, *Z* = 2,000,000 random sequences are generated, which get mutated at a random number of 1 to *n* positions of design shell residues to randomly chosen amino acid residues and are fed into the NN to compute their score $RS_{\mathrm{NN}}^{j}$. The dataset PRED_*r*_ contains the *Z* sequences *des*_*seq*_*j*,*r*_ ranked according to their $RS_{\mathrm{NN}}^{j}$ values. For the *s*/2 best scoring candidate sequences *cand*_*r*_, the chosen Rosetta scoring function is used to compute their $RS_{3\mathrm{DM}}^{j}$ values. To prepare the set OPT_*r*+1_ utilized in the next iteration, Rosetta:MSF:NN replaces the bottom half of the OPT_*r*_ sequences with the *s*/2 candidates *cand*_*r*_. These iterations continue until a user-defined stopping criterion is satisfied. As Rosetta:MSF:NN was utilized for an MSD protocol, a separate NN was trained for each individual state of each protein.

### Assessing design performance

To determine the score of a candidate sequence *des*_*seq*_*j*_ for an MSD protocol, the mean Rosetta score was computed:

$$RS_{3\mathrm{DM}}^{j}(des\_seq_{j})=\frac{1}{m}\sum_{i=1}^{m}ts_{i}(des\_seq_{j})$$ (1)

Here, *m* is the number of states and *ts*_*i*_(*des*_*seq*_*j*_) is the Rosetta total score for a sequence given a state *i*. In all equations, Rosetta scores are indicated in REUs.

To assess the fitness of a sequence set OPT_*r*_ of an iteration *r*, the mean of all *s* = 239 $RS_{3\mathrm{DM}}^{j}(des\_seq_{j})$ values was determined:

$$RS({\mathrm{OPT}}_{r})=\frac{1}{s}\sum_{j=1}^{s}RS_{3\mathrm{DM}}^{j}(des\_seq_{j,r}^{})$$ (2)

To distinguish the values related to the NN- and GA-based protocol, they were designated *RS*_NN_(OPT_*r*_) and *RS*_GA_(OPT_*r*_), respectively.

For the determination of the design-specific normalized areas above (NAA) the *RS*_NN_(OPT_*r*_) and *RS*_GA_(OPT_*r*_) values, the areas flanked by the specific *RS*(OPT_1_) and *RS*(OPT_100_) values were calculated. For their normalization between 0.0 and 1.0, the lowest value reached after 100 generations by either of the two protocols was used.

### Following trends of sequence sampling

To characterize trends of sequence sampling, for each design *prot*_*k*_ of MD_EnzBench the composition of the sequence sets ${\mathrm{OPT}}_{k,r}^{\mathrm{NN}}$ (*r* = 1–100) generated by the NN-based protocol was compared with a well-defined reference sequence set ${\mathrm{OPT}}_{k,\delta^{*}}^{\mathrm{GA}}$.

To begin with, for each design *prot*_*k*_ the reference set ${\mathrm{OPT}}_{k,\delta^{*}}^{\mathrm{GA}}$ was identified. This set represents among the first *δ* = 1−500 GA iterations the earliest generation δ*, whose score value was most similar to $RS_{\mathrm{NN}}^{}{(\mathrm{OPT}}_{k,100}^{\mathrm{NN}})$, which was generated by Rosetta:MSF:NN:enzdes during the last iteration of the NN-based protocol. Utilizing the amino acid composition of the related 239 sequences *des*_*seq*_*j*,*r*_ belonging to ${\mathrm{OPT}}_{k,\delta^{*}}^{\mathrm{GA}}$, a normalized frequency table $ft_{k,pos}^{\mathrm{GA}}=f_{k,pos}^{\mathrm{GA}}(aa_{i})(i=1-20)$ was deduced for each of the *n* residue positions *pos* of the design shells. Analogously, frequency tables $ft_{k,pos,r}^{\mathrm{NN}}$ were derived from the 100 ${\mathrm{OPT}}_{k,r}^{\mathrm{NN}}$ sets created during all iterations of the NN-based protocol. Position-specific Euclidean distances $euk_{r}(ft_{k,pos,r}^{\mathrm{NN}},ft_{k,pos}^{\mathrm{GA}})(r=1-100)$ were computed according to:

$$euk_{r}(ft_{k,pos,r}^{\mathrm{NN}},ft_{k,pos}^{\mathrm{GA}})=\sqrt[{}]{\sum_{i=1}^{20}{\left(f_{k,pos,r}^{\mathrm{NN}}(aa_{i}^{})-f_{k,pos}^{\mathrm{GA}}(aa_{i}^{})\right)}^{2}}$$ (3)

To assess the mean amino acid variation for each iteration *r* and each design *k*, the Euclidean distances were averaged according to:

$$dist_{k,r}=\frac{1}{n}\sum_{pos=1}^{n}euk_{r}(ft_{k,pos,r}^{\mathrm{NN}},ft_{k,pos}^{\mathrm{GA}})$$ (4)

*dist*_*k*,*r*_ values were divided in two groups ${\mathrm{NN}}_{1}^{k}{=\{\mathrm{dist}}_{k,r}^{}|r=1-50\}$ and ${\mathrm{NN}}_{2}^{k}{=\{\mathrm{dist}}_{k,r}^{}|r=51-100\}$ to distinguish the composition of the sequences generated during the first and second half of the iterations.

To compute “null model” distributions $R_{1}^{k}$ and $R_{2}^{k}$ that served as references, the frequencies were shuffled table-wise for each of the 100×*n* $ft_{k,pos,r}^{\mathrm{NN}}$ sets. Afterwards, Euclidean distances to $ft_{k,pos}^{\mathrm{GA}}$ were computed according to Eq 3 and their mean was determined according to Eq 4. The four *prot*_*k*_ specific distributions of *dist*_*k*,*r*_ values were visualized by means of a box plot.

## Results and discussion

### A 4-layer NN is able to approximate the energy landscape of a design problem

Our basic assumption was that an NN can deduce the energy landscape of a design problem, if it is trained with a small number of sequences, whose design-specific Rosetta scores are known. In order to test this hypothesis, we had to choose a suitable representation of amino acid residues, the number of residues fed into the NN, and a network topology. We decided to represent each amino acid residue *aa* by means of one-hot encoding. The amino acid specific, 20-dimensional vectors are listed in **S1 Table**. We restricted the number of residue positions fed into the NN to those *n* ones that are subjected to the design process, i.e. belong to the design shell. To deduce a prediction of the Rosetta score from these 20*n* features, we opted for a 4-layered, fully connected, feed-forward network. We chose two hidden layers, because these feed-forward networks generalize better than those with only one hidden layer [40]. As we expected mutual dependencies in the occupancy of the structurally adjacent positions of design shells, we opted for a fully connected architecture, because it is capable of learning any function [41]. The representation of the candidate sequences proposed an input layer consisting of 20*n* neurons and an output layer consisting of one neuron that presents the predicted score as a real value. Based on a grid search for the number of hidden neurons (**S1 Fig**), we chose a first hidden layer with 10+20*n*/2 neurons and a second hidden layer with 5+20*n*/4 neurons; see **Fig 1A**.

For a first assessment of the predictive power of the NN, we utilized the outcome of a comprehensive Rosetta:MSF:GA:enzdes run of a HisB-N design, which was based on a genetic algorithm (GA). Briefly, a GA imitates the process of natural selection by maintaining a population of design sequences that are evolving for several generations. The MSD protocol Rosetta:MSF:GA generates candidate sequences by using the well-proven GA of Rosetta and computes their Rosetta total score averaged over all chosen states; for details see [15] and references therein. MSD is superior over SSD even for seemingly simpler design task: Compared to SSD, a positive MSD approach similar to the one used here has more clearly identified for a dataset of 15 design problems the sequences that are optimal for all states [23].

These HisB-N data are from an on-going design project, where we want to accommodate a new ligand in the binding site of the enzyme HisB-N by altering the occupancy of *n* = 14 residue positions. During 500 iterations of the GA, the Rosetta scores *RS*_3DM_ of 48,588 candidate sequences were computed, each based on a sequence-specific 3D_opt_ model. This set, which we named HisB_GA_raw_ consisted of tuples $des\_seq_{j}^{raw}=(aa_{1}^{j},..,aa_{pos}^{j},..,aa_{14}^{j},RS_{3\mathrm{DM}}^{j})$, where each 20-dimensional vector $aa_{pos}^{j}$ represents one amino acid at a position *pos* of a candidate sequence *j* and $RS_{3\mathrm{DM}}^{j}$ is the corresponding and normalized score (Eq 1) deduced by Rosetta from specific 3D_opt_ models. We randomly selected 67% of the HisB_GA_raw_ tuples and used them to train the NN; for details see Materials and methods. After training, we utilized this NN to determine predicted Rosetta scores $RS_{\mathrm{NN}}^{j}$ for the remaining 33% of the feature vectors $des\_seq_{j}^{raw}=(aa_{1}^{j},\ldots ,aa_{14}^{j})$.

**Fig 2:**
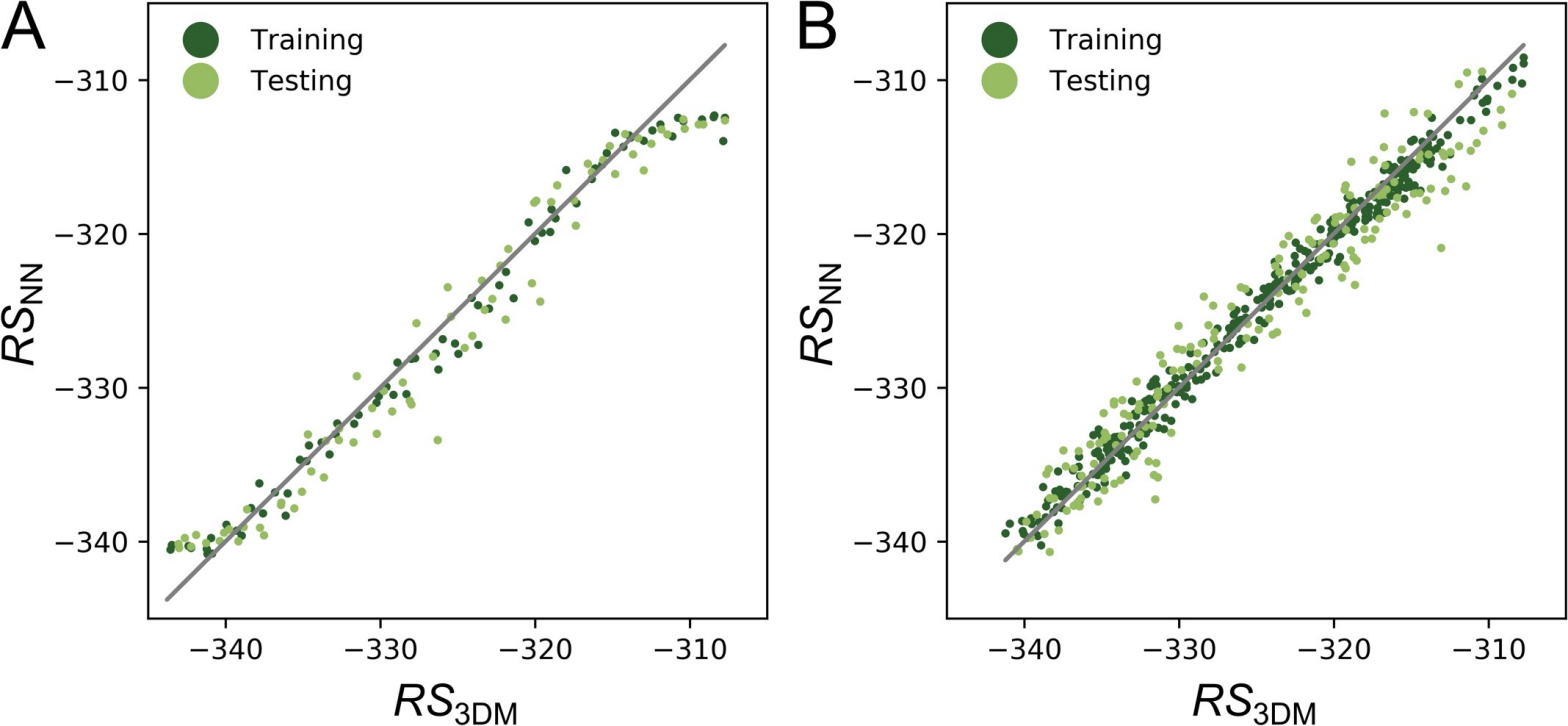
Performance of NNs for sequence sets with different sequence similarities. In both panels, Rosetta scores $RS_{3\mathrm{DM}}^{j}$ deduced from 3D_opt_ models are plotted versus the Rosetta scores $RS_{\mathrm{NN}}^{j}$ predicted by NNs for an enzyme design. Scores are given in REUs and training and test data are represented as dark and light green dots, respectively. The gray lines indicate the diagonal, i.e. the position of perfect predictions. (A) Performance resulting for the dataset HisB_GA_raw_ that consists mainly of sequences with point mutations. The PCC deduced from $RS_{3\mathrm{DM}}^{j}$ and $RS_{\mathrm{NN}}^{j}$ values of the test data was 0.95. For reasons of clarity, a large number of overlapping dots were removed; the plot shown in **S2A Fig** contains the full dataset. (B) Performance resulting from the redundancy-free dataset HisB_GA_nr_ that consists of 533 sequences with a maximal pairwise sequence identity of 70%. The PCC was 0.97.

In **Fig 2A**, $RS_{\mathrm{NN}}^{j}$ values are plotted versus the corresponding $RS_{3\mathrm{DM}}^{j}$ values. The clearly visible correspondence of both scores is evidenced by the high value of the Pearson correlation coefficient (PCC), which was 0.95 (*p* << 1E-100) for the set of all test data. The average error determined for the test dataset was not larger than 0.96 Rosetta Energy Units (REU). This test set was generated by the GA and of the 16,196 sequences, not more than 110 contain more than two mutations. Thus, this dataset did not allow us to study whether the NN is able to correctly score more strongly deviating sequences.

In order to assess the predictive performance of the NN for more difficult cases, we utilized HisB_GA_raw_ to deduce the non-redundant dataset HisB_GA_nr_. By accepting a maximal pairwise sequence identity of 70%, 533 tuples were identified that differed at least in four residue occupancies. The set HisB_GA_nr_ was used to train and test the NN as described above. As **Fig 2B** shows, the NN performed well also for the more difficult cases: The PCC was 0.97 (*p* < 1E-100) and the average error was 1.8 REU.

To generate the plot shown in **S2B Fig**, we exclusively utilized the 238 HisB_GA_raw_ tuples generated during the first iteration of the genetic algorithm. For the 80 test cases, the PCC dropped to 0.62 (*p* = 1.2E-9) and the average error was 2.9 REU. These values indicate that one iteration of the GA is not sufficient to sample the complex energy landscape of this design problem in an adequate manner.

Taken together, we concluded that the representation of amino acid side chains with one-hot encoding and a trained NN are appropriate to model that part of the energy landscape (Rosetta scores) sampled by a GA during the problem-specific enzdes calculation.

### Rosetta:MSF:NN, a hybrid framework for MSD

The training with the HisB_GA_raw_ tuples generated during the first GA iteration was too sparse for the complex energy landscape to be learnt by the NN. **S2B Fig** suggests that the $RS_{3\mathrm{DM}}^{j}$ values of several rounds of sequence optimization are required for an adequate representation of this energy landscape. However, one cannot predefine the number of iterations needed to find optimal sequences for a given design problem, as convergence speed is problem-specific. Guided by this constraint, we designed a novel protocol for sequence search; compare **Fig 1B**. We decided to replace the genetic algorithm of Rosetta:MSF:GA with an NN-based sequence selection, but to continue keeping a specific set OPT_*r*_ = {*des*_*seq*_*j*,*r*_|1≤*j*≤*s*} of *s* optimal sequences for each iteration *r*. Rosetta:MSF:NN computes the $RS_{3\mathrm{DM}}^{j}$ score based on sequence-specific 3D_opt_ models only for newly generated elements of OPT_*r*_. The union of all OPT_*t*_ tuples determined during the preceding iterations *t* = 1, …, *r*-1 constitutes the continuously growing training set ${\mathrm{OPT}}_{r}^{*}$, which is used to re-train the NN for iteration *r*.

The re-trained NN is then utilized to predict the scores $RS_{\mathrm{NN}}^{j}$ of an extensive number *Z* of randomly generated sequences; the default value is *Z* = 2,000,000. For those *s*/2 sequences with highest $RS_{\mathrm{NN}}^{j}$ values, Rosetta:MSF:NN computes the $RS_{3\mathrm{DM}}^{j}$ values based on sequence-specific 3D_opt_ models; for details see Materials and methods. To compile the next set OPT_*r*+1_, half of the *des*_*seq*_*j*,*r*_ tuples are replaced with newly generated ones and their corresponding $RS_{3\mathrm{DM}}^{j}$ values. Analogously to Rosetta:MSF:GA, the user has to execute the novel algorithm for several iterations until convergence is reached. This hybrid algorithm of sequence selection combines three major advantages:

1. Due to the high speed of the NN in computing $RS_{\mathrm{NN}}^{j}$ values, an extensive number of candidate sequences can be assessed, and the $RS_{\mathrm{NN}}^{j}$ values approximate the $RS_{3\mathrm{DM}}^{j}$ values quite well. The determination of $RS_{3\mathrm{DM}}^{j}$ values for all possible candidate sequences is currently not feasible due to the computational costs of the 3D_opt_ models.
2. By merging the iteratively generated sets OPT_*r*_ that consist of all hitherto found well-scoring sequences, the prediction quality of the NN increases continuously due to the denser sampling of the problem-specific energy landscape.
3. The iteration-specific determination of $RS_{3\mathrm{DM}}^{j}$ values for the sequences with highest $RS_{\mathrm{NN}}^{j}$ values provides a corrective feedback and helps to eliminate less optimal NN predictions.

Encouraged by these promising initial results, we wanted to answer the following four questions in order to assess the potential benefit of integrating an NN into Rosetta:MSF:

1. Does the use of NNs reduce the number of iterations needed to identify optimal candidate sequences?
2. How robust is this approach with respect to the chosen features and scoring functions?
3. Does the NN-based approach find sequences with better Rosetta scores?
4. Does the extensive sampling of sequence space lead to candidate sequences not found by the GA?

### Rosetta:MSF:NN converges 10-times faster than Rosetta:MSF:GA and enumerates better scoring sequences

For a comprehensive comparison of Rosetta:MSF:NN with the older Rosetta:MSF:GA protocol, we utilized the previously introduced benchmark MD_EnzBench [15]. This set has been compiled to test the ability of protocols to rebuild the ligand-binding site of 16 proteins *prot*_*k*_. For each *prot*_*k*_, 10 specifically prepared conformations that served as states of an MSD protocol have been deduced by means of molecular dynamics simulations. Each conformation contains the bound ligand and in order to increase the difficulty of the design task, all residues of the design shells were replaced with alanines; for details see Materials and methods.

**Fig 3:**
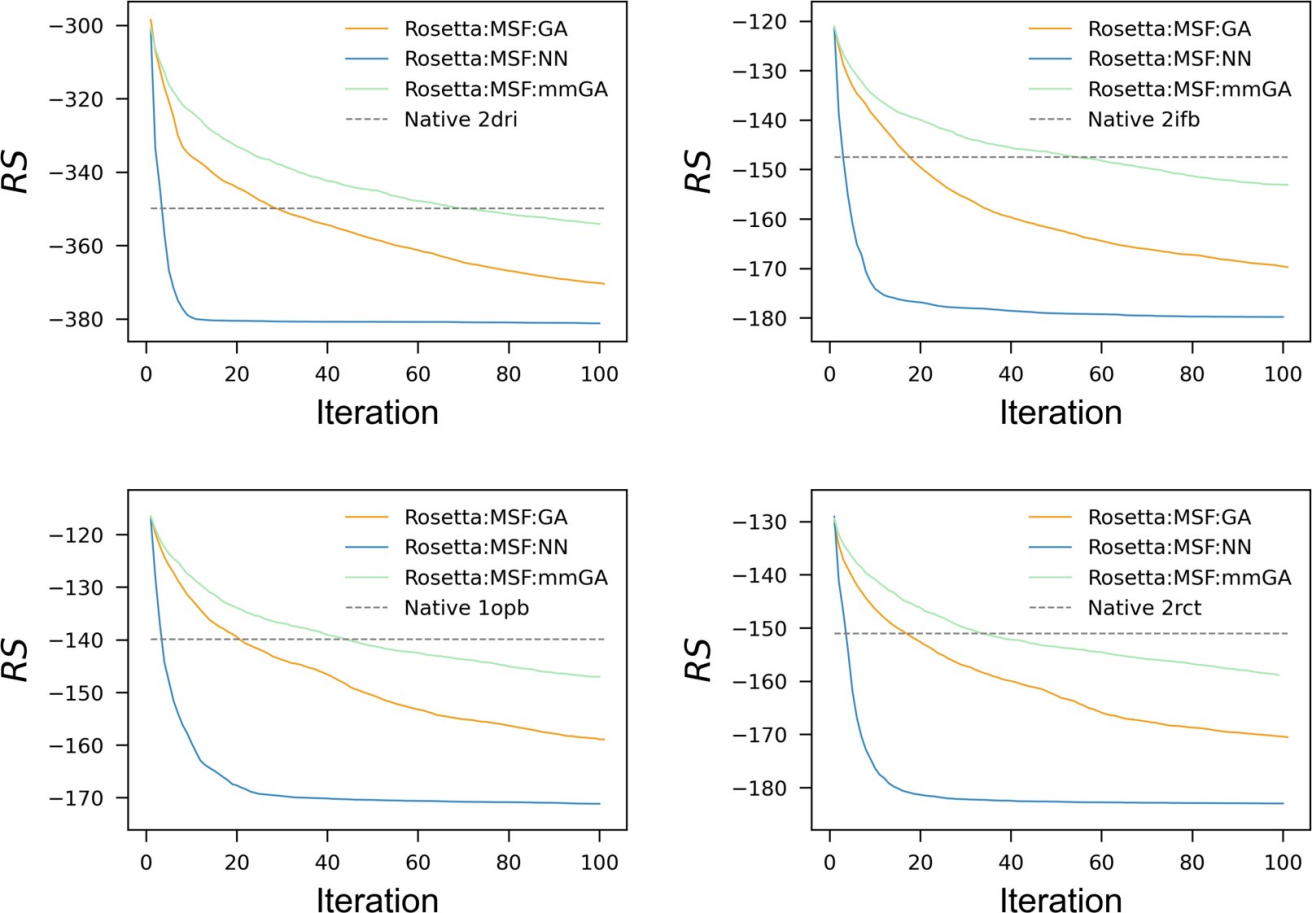
Convergence of Rosetta:MSF:GA:enzdes, Rosetta:MSF:mmGA:enzdes, and of Rosetta:MSF:NN:enzdes. The three protocols were utilized for each of the four designs from *repr_prot*. The mean Rosetta scores *RS*(OPT_*r*_) were determined in REUs for each iteration *r =* 1–100 and plotted. The blue lines represent the *RS*_NN_(OPT_*r*_) values, the orange lines the *RS*_GA_(OPT_*r*_) and the green line the *RS*_mmGA_(OPT_*r*_) values. The dashed horizontal line marks the score of the relaxed native protein; the PDB-ID of the corresponding native protein is indicated. The Rosetta:MSF:GA:enzdes and Rosetta:MSF:NN:enzdes plots for all 16 designs are shown in S3 Fig. mmGA is a genetic algorithm that introduces multiple mutations.

For these 16 design problems, Rosetta:MSF:GA:enzdes and Rosetta:MSF:NN:enzdes were executed as 10-state MSD protocols for at least 100 iterations and the NNs were re-trained during each iteration as described. In order to follow the convergence of the two design processes, the mean Rosetta score *RS*(OPT_*r*_) (Eq 2) was determined for each iteration *r*. *RS*(OPT_*r*_) is the mean of the $RS_{3\mathrm{DM}}^{j}$ scores of all sequences related to the iteration-specific set OPT_*r*_. In **Fig 3**, the protocol-specific convergence is shown for four representative examples *repr*_*prot* = {2dri,2ifb,lopb,2rct}, which are indicated by their PDB-ID, and all 16 designs are documented in **S3 Fig**. As can be seen, both protocols find sequences that score considerably better than the native ones. Most interestingly, the *RS*_NN_(OPT_*r*_) values dropped more rapidly than the *RS*_GA_(OPT_*r*_) values. In order to assess the momentum of convergence numerically, we determined the normalized area above (NAA) the *RS*_NN_(OPT_*r*_) and the *RS*_GA_(OPT_*r*_) values in analogy to a ROC curve [42]. An NAA value gets close to 1.0, if the curve drops vertically to its minimum during the first few iterations. In **Table 1**, the NAA_NN_ and NAA_GA_ values are listed for all 16 designs as well as the numbers *n* and *ss* of design shell residues and second shell residues; for their definition see [8]. The comparison of the design-specific NAA_NN_ and NAA_GA_ values confirmed that Rosetta:MSF:NN:enzdes converged much faster than the GA-based protocol: The mean *RS*_NN_(OPT_*r*_) value was 0.95, whereas the mean *RS*_GA_(OPT_*r*_) value was 0.63. Although trained with relatively few examples during the first iterations, Rosetta:MSF:NN:enzdes found more rapidly sequences with low Rosetta scores than the GA-based protocol did.

**Table 1:** Performance comparison of the GA- and NN-based protocols.

PDB-ID	*n*	*ss*	NAA_GA_	NAA_NN_	$r_{k}^{*}$	$r_{k,\mathrm{GA}}^{\mathrm{native}}$	$r_{k,\mathrm{NN}}^{\mathrm{native}}$
1fzq	20	29	0.59	0.96	8	32	4
1hsl	19	42	0.73	0.96	9	16	3
1j6z	27	45	0.75	0.95	9	31	5
1n4h	25	51	0.62	0.95	7	19	3
1nq7	28	57	0.62	0.95	9	24	4
1opb	22	50	0.57	0.93	10	21	4
1pot	19	41	0.67	0.94	7	4	2
1urg	19	40	0.56	0.96	7	24	3
2b3b	17	46	0.68	0.97	6	25	3
2dri	19	42	0.67	0.97	6	29	4
2ifb	22	54	0.63	0.95	8	18	3
2q2y	23	34	0.67	0.96	8	47	5
2qo4	22	40	0.58	0.96	7	25	4
2rct	22	51	0.57	0.94	7	17	4
2rde	20	35	0.61	0.93	7	19	4
2uyi	23	35	0.61	0.96	7	61	5
**Average**	**22**	**43**	**0.63**	**0.95**	**7.6**	**25.8**	**3.8**

All values were determined for each of the *k* = 16 proteins (indicated by their PDB-ID) from the MD_EnzBench set. *n* is the number of design shell residues and *ss* the number of second shell residues, respectively. The NAA_GA_ and NAA_NN_ values specify the area above the plots of the corresponding *RS* values for the GA- and NN-based protocol. $r_{k}^{*}$ is the number of the first NN iteration, whose *RS*_NN_(OPT_*r*_) value reached the *RS*_GA_(OPT_100_) value that served as a reference. The $r_{k,\mathrm{GA}}^{\mathrm{native}}$ and $r_{k,\mathrm{NN}}^{\mathrm{native}}$ values indicate for the GA- and the NN-based protocols the number of the first iteration that generated a sequence set having native-like Rosetta energies.

To estimate the performance gain, we determined for each design *k* the first iteration $r_{k}^{*}$, whose *RS*_NN_(OPT_*r*_) value reached the *RS*_GA_(OPT_100_) value of iteration 100 of Rosetta:MSF:GA:enzdes. As **Table 1** shows, the $r_{k}^{*}$ values varied between 6 and 10; this spread reflects most likely the differing complexity of the design-specific energy landscapes. On average, 7.6 iterations of the NN-based protocol were sufficient to find sequences that scored as good as those created by the GA-based protocol in iteration 100. We concluded that Rosetta:MSF:NN:enzdes converges approximately ten times faster than the GA-based protocol. A sevenfold gain results, if one compares for both approaches the first iterations $r_{k,\mathrm{GA}}^{\mathrm{native}}$ and $r_{k,\mathrm{NN}}^{\mathrm{native}}$ that generated a sequence set OPT_*r*_ having native-like Rosetta energies. On average, the GA needed 25.8, and the NN approach not more than 3.8 iterations; compare **Table 1**. Interestingly, convergence speed seems uncorrelated with the number of the design shell residues; of the three Spearman rank order correlation tests of *n* with NAA_NN_, NAA_GA_, and $r_{k}^{*}$, none gave a statistically significant result.

The comparison of the NAA_NN_ and NAA_GA_ values and the plots shown in **S3 Fig** indicate that Rosetta:MSF:NN:enzdes finds for a given number of iterations better-scoring sequences than the GA-based protocol. If both protocols were executed for 100 iterations, the *RS*_NN_(OPT_100_) value was superior to the *RS*_GA_(OPT_100_) value in all designs; compare **S3 Fig**. These findings confirm that the NN can beneficially exploit the extensive sampling to identify sequences with superior Rosetta scores.

The notably reduced number of iterations that are sufficient for Rosetta:MSF:NN:enzdes to reach a minimum, leads to time savings, if one condition is met: The protocol is faster, if the extra time required to train and use the NN has no drastic effect on the computing time needed to create a new generation of sequences. In order to estimate runtimes, we compared the performance of the GA- and the NN-based approach for the HisB design. For a fair comparison, execution times were determined for designs running on one CPU.

Most expensive was the determination of the $RS_{3\mathrm{DM}}^{j}$ scores and Rosetta:MSF:GA:enzdes required constantly 32 h for each iteration. In contrast, the execution time of Rosetta:MSF:NN:enzdes increases linearly with the size of the training set, which grows due to the union of the OPT_*r*_ sets. During the first iteration, two minutes were required for training and approximately 15 minutes for the determination of the new candidate sequences. For generation *r*, execution time *t*(*r*) accumulated to $t(r)=r\cdot (32h+10\min)+\sum_{s=1}^{r}(s\cdot 2\min)$.

This minor increase of the iteration-specific execution time is more than compensated by the reduced number of iterations: As **Table 1** shows, Rosetta:MSF:NN:enzdes needs on average only eight iterations to find sequences having scores comparable to those from generation 100 of Rosetta:MSF:GA:enzdes. Thus, for a design task whose complexity is comparable to the HisB case, the GA consumes approximately 3200 h to finish 100 iterations. In contrast, the NN-based approach needs not more than 8 iterations, which are finished after 256 h + 80 min + 72 min, i.e. after less than 260 h. This approximation makes clear that the faster convergence of Rosetta:MSF:NN:enzdes has a drastic effect on runtime due to the relatively minor expense added by the NN.

In principle, the NN-based protocol is applicable to all design algorithms that fulfill two prerequisites: i) The algorithm can provide tuples $des\_seq_{j}^{}=(aa_{1}^{j},..,aa_{pos}^{j},..,aa_{n}^{j},3D-score^{j})$ each consisting of a sequence and the corresponding 3*D*−*score*^*j*^. ii) The algorithm can process the NN output, which is a list of newly generated *des*_*seq*_*j*_ candidates. The NN-based protocol is most useful, if the calculation of approximative $3D-score_{approx}^{j}$ values reduces execution time, which has to be determined for each application. For example, protein design by means of EvoEF2 [43] or FoldX [44] is relatively fast: EvoEF2 is capable of designing de novo proteins consisting of 200 residues in 15 min and FoldX is at most five times slower [43]. Thus, the additional expense for training and using an NN does not seem justified for these applications.

### Rosetta:MSF:NN performs well with different representations of amino acid residues, scoring functions, and design protocols

In order to assess the robustness and the applicability of Rosetta:MSF:NN, we performed additional redesign experiments of the four proteins from the set *repr_prot*. First, we wanted to make plausible that the performance of Rosetta:MSF:NN does not critically depend on the chosen one-hot encoding of residues shown in **S1 Table**. For the experiment named NN_FT, residues were not represented by one-hot encoding, but by vectors indicating the five features “volume”, “polarity”, “isoelectric point”, “hydrophobicity”, and “mean solvent accessibility” [45]; compare **S2 Table**. The analysis of the plots shown in **S4 Fig** reveals that this alternative residue representation does not reach the performance of one-hot encoding, but performs better than the GA: In all cases, the NN-based algorithm converged faster than the GA-based one.

For all designs presented so far, we utilized the REF15 scoring function [46]. In order to make plausible that the convergence of Rosetta:MSF:NN is not dependent on a specific scoring system, another four redesign experiments were performed for the proteins from *repr_prot* by using the alternative *talaris* [47] scoring function: A clear convergence gain of the NN- over the GA-based protocol is confirmed by **S5 Fig** for the *talaris* scoring function as well.

The FastDesign (FD) protocol iterates between sequence optimization and structure refinement and has been used to design novel protein folds and assemblies [48], but also for enzyme design [49]. For an initial assessment of the NN’s ability to find proper sequences in the FD-specific energy landscape, we applied the settings “FastDesign (with ligand)” introduced recently [49] to the four proteins from *repr_prot*. For a comprehensive sampling of the energy landscape and an initial training of the NN, we used FastDesign to enumerate for each protein 1000 sequences ${\mathrm{FD}}_{1000}^{}=des\_seq_{j=1-1000}^{\mathrm{FD}}=(aa_{1}^{j},..,aa_{pos}^{j},..,aa_{n}^{j},RS_{\mathrm{FD}}^{j})$, which is more than twice of the designs used elsewhere [49]. As **Table 2** indicates, the extensive sampling has no drastic effect on the scores: The mean of all 1000 $RS_{\mathrm{FD}}^{j}$-values and of the 250 best ones (FD_best250_) differed at most by 6.5 REU. As already observed with the enzdes protocol, the native sequences were scored worse than the designs. The $RS_{\mathrm{FD}}^{\mathrm{native}}$ values of 2dri, 2ifb, 1opb, and 2rct were -894.4, -391.1, -419.5, and -434.8, respectively.

**Table 2:** Performance of FastDesign and a protocol based on four iterations of NN training.

	2dri		2ifb		1opb		2rct	
	*RS*_FD_	$dist_{r}^{\mathrm{FD}}$	*RS*_FD_	$dist_{r}^{\mathrm{FD}}$	*RS*_FD_	$dist_{r}^{\mathrm{FD}}$	*RS*_FD_	$dist_{r}^{\mathrm{FD}}$
FD_1000_	-929.3	-	-407.6	-	-438.0	-	-459.0	-
FD_best250_	-934.0	-	-412.7	-	-442.7	-	-465.3	-
NN(FD_1000_)	-880.0	0.50	-386.0	0.49	-391.1	0.46	-435.4	0.51
${\mathrm{OPT}}_{1}^{\mathrm{FD}}$	-929.8	0.03	-407.7	0.04	-438.0	0.04	-459.1	0.05
FD_NN_1_	-889.4	0.51	-370.4	0.43	-404.1	0.48	-413.7	0.52
FD_NN_2_	-897.8	0.38	-382.4	0.37	-385.8	0.55	-432.6	0.34
FD_NN_3_	-924.8	0.38	-400.0	0.43	-437.5	0.38	-448.5	0.39
FD_NN_4_	-933.2	0.50	-410.8	0.56	-442.2	0.50	-457.7	0.41

The *RS*_FD_ columns list mean FastDesign scores and the $dist_{r}^{\mathrm{FD}}$ columns frequency distances for designs with the four proteins from *repr_prot*. Rows FD_1000_ and FD_best250_ represent the values related to 1000 and the best 250 FastDesign sequences. The NN(FD_1000_) values resulted from an NN training with the FD_1000_ sequences. The ${\mathrm{OPT}}_{1}^{\mathrm{FD}}$ values represent the initial training set of an iterative training that generated the output FD_NN_1_ to FD_NN_4_.

For each protein, the 1000 sequences and their $RS_{\mathrm{FD}}^{j}$-values were used to train an NN. The trained NNs were utilized to score *Z* = 2,000,000 randomly generated sequences and in analogy to our other comparisons, those 250 ones possessing the highest $RS_{\mathrm{NN}}^{j}$-values were selected. To assess the quality of the NN predictions, these sequences were scored by means of FastDesign and the mean values were determined. As **Table 2** indicates, the NN(FD_1000_) approach, i.e. a singular training of an NN with 1000 optimal cases performed poorly: In all four cases, the mean values were more than 25 REU worse than the mean FD_best250_ values.

Most plausibly, the poor performance is (also in this case) due to the lack of unsuitable sequences in the training set. As a compensation, we applied the following protocol that iterates between FastDesign steps and NN-based sequence predictions: Initially, FastDesign was used to generate 250 sequences, which served in combination with their FastDesign scores $RS_{\mathrm{FD}}^{j}$ as training cases ${\mathrm{OPT}}_{1}^{\mathrm{FD}}=des\_seq_{j=1-250}^{\mathrm{FD}}=(aa_{1}^{j},..,aa_{pos}^{j},..,aa_{n}^{j},RS_{\mathrm{FD}}^{j})$. After training, the NN was used to generate *Z* = 2,000,000 sequences and those 250 ones with highest $RS_{\mathrm{NN}}^{j}$-values were scored by means of FastDesign and stored as the result FD_NN_1_. The union of this sequence set and of ${\mathrm{OPT}}_{1}^{\mathrm{FD}}$ gave the training set ${\mathrm{OPT}}_{2}^{\mathrm{FD}}$. During the second iteration, the NN was trained with these 500 sequences and used to create 250 sequences constituting FD_NN_2_. For these sequences, their $RS_{\mathrm{FD}}^{j}$ score was determined and they were merged with ${\mathrm{OPT}}_{2}^{\mathrm{FD}}$ to the training set ${\mathrm{OPT}}_{3}^{\mathrm{FD}}$. During a third and a fourth iteration, the NN was trained with 750 ${\mathrm{OPT}}_{3}^{\mathrm{FD}}$ and 1000 ${\mathrm{OPT}}_{4}^{\mathrm{FD}}$ sequences, and the two sets FD_NN_3_ and FD_NN_4_ were generated, each consisting of 250 sequences and their $RS_{\mathrm{FD}}^{j}$ scores.

The iteration-specific mean *RS*_FD_ values shown in **Table 2** confirm that this approach performed much better than the single-iteration training with 1000 sequences: For three proteins, the mean FD_NN_4_ value differed not more than 2 REU from the FD_best250_ value and the maximal difference was 7.6 REU. These findings suggest for most cases that the corrective feedback generated during a minimum of four training iterations is sufficient for an NN to learn the FastDesign specific energy landscape. Iterative training is more effective, as the newly computed *RS*_FD_ values provide valuable feedback and allow the NN to avoid false positive predictions.

In summary, these results emphasize the capability of the NNs to learn a problem-specific energy landscape modelled by means of different residue representations, scoring functions, and protocols.

### The extensive sampling of the sequence space finds alternative minima

With default values, our NN-based approach allows per iteration the assessment of 2,000,000 alternative sequences; in contrast, the GA-based approach generates less than 120 novel candidates per generation. We were interested to find out, whether this rigorous widening of search space sampling had a pronounced effect on the composition of the enzdes outcome. It is difficult to compare precisely the composition of two sequence sets, thus we opted for an approximate approach, namely the comparison of amino acid frequency tables.

To characterize trends, we wanted to compare for each design *prot*_*k*_ of MD_EnzBench the composition of the sequence sets ${\mathrm{OPT}}_{k,r}^{\mathrm{NN}}$ (*r* = 1–100) generated by the NN-based protocol with a fixed reference sequence set ${\mathrm{OPT}}_{k,\delta^{*}}^{\mathrm{GA}}$ consisting of sequences generated by a late iteration of the GA-based protocol. If both protocols sample the same area of sequence space, the amino acid composition of the NN-based outcome should iteratively approach the amino acid frequencies of the GA-based reference.

**Fig 4:**
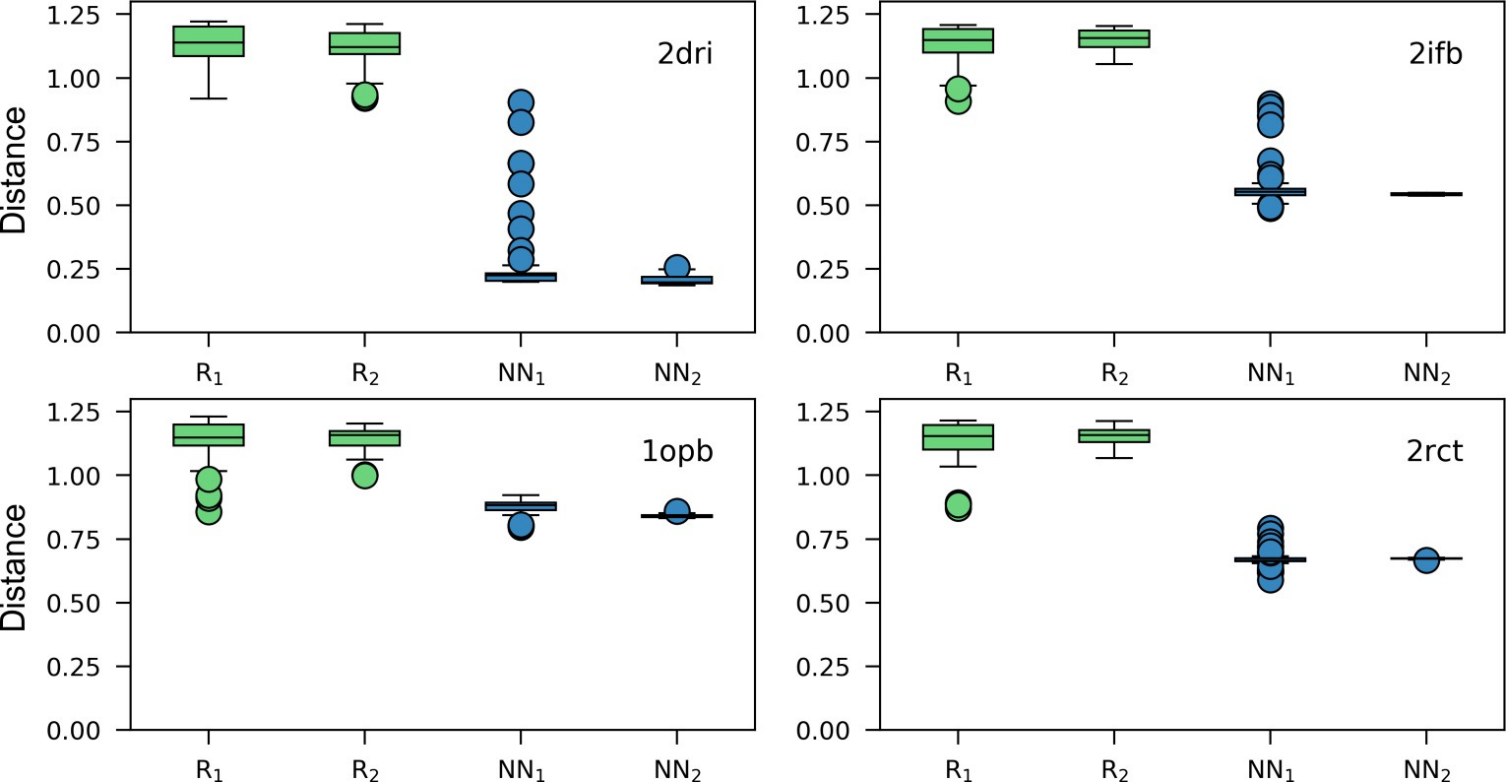
Amino acid frequency distributions for the outcome of the first and second half of design protocols. The boxplots represent the distributions of mean distances between amino acid frequencies tables *ft*_*k*,*pos*,*r*_ related to the iterations of the NN-based protocol and a reference table $ft_{k,pos}^{\mathrm{GA}}$ from the GA-based protocol; compare Eq 3 and Eq 4. The group ${\mathrm{NN}}_{1}^{k}$ contains the distributions related to the first 50 and ${\mathrm{NN}}_{2}^{k}$ the distributions of the second 50 iterations. For the computation of $R_{1}^{k}$ and $R_{2}^{k}$, the values of each table $ft_{k,pos,r}^{\mathrm{NN}}$ were shuffled; for details see Materials and methods. The boxplots show the results for the four designs with the set *repr_prot*.

To begin with, we determined for each design *prot*_*k*_ of MD_EnzBench the set ${\mathrm{OPT}}_{k,\delta^{*}}^{\mathrm{GA}}$ among *δ* = 1,…,500 GA iterations. It belonged to the first generation δ* whose sequence set ${\mathrm{OPT}}_{k,\delta }^{\mathrm{GA}}$ had a score comparable to that of the ${\mathrm{OPT}}_{k,100}^{\mathrm{NN}}$ set. In order to estimate compositional differences, we deduced for each of the *n* design shell residue positions *pos* a reference frequency table $ft_{k,pos}^{\mathrm{GA}}=f_{k,pos}^{\mathrm{GA}}(aa_{i})(i=1-20)$ from ${\mathrm{OPT}}_{k,\delta^{*}}^{\mathrm{GA}}$ and further frequency tables $ft_{k,pos,r}^{\mathrm{NN}}$ from all of the 100 ${\mathrm{OPT}}_{k,r}^{\mathrm{NN}}$ sets. Euclidean distances $euk_{r}(ft_{k,pos,r}^{\mathrm{NN}},ft_{k,pos}^{\mathrm{GA}})(r=1-100)$ (Eq 3) were computed and their mean *dist*_*k*,*r*_ determined according to Eq 4. In order to assess the progression of compositional differences, these were divided into two groups ${\mathrm{NN}}_{1}^{k}{=\{\mathrm{dist}}_{k,r}^{}|r=1-50\}$ and ${\mathrm{NN}}_{2}^{k}{=\{\mathrm{dist}}_{k,r}^{}|r=51-100\}$ consisting of the outcome of the first and second half of the iterations. In order to generate “null model” distributions $R_{1}^{k}$ and $R_{2}^{k}$, the $ft_{k,r}^{\mathrm{NN}}$ frequencies were shuffled prior to the computation of their Euclidean distances to $ft_{k}^{\mathrm{GA}}$. For each design *prot*_*k*_ these four distributions of distances were visualized by means of a box plot. **Fig 4** shows that in all four cases from *repr_prot*, the mean of the distances sampled with the two null models $R_{1}^{k}$ and $R_{2}^{k}$ was close to the maximally possible distance, which is $\sqrt{2}\approx 1.4$. All ${\mathrm{NN}}_{1}^{k}$ and ${\mathrm{NN}}_{2}^{k}$ distributions had lower, but substantial distances to $ft_{k}^{\mathrm{GA}}$. The sequences found for 2dri by the GA- and NN-based protocols resemble each other to a certain extent (mean 0.2), but those found for the other three designs are more dissimilar (mean > 0.56). This finding suggests that the GA- and the NN-based protocols concentrate on different regions of sequence space. In all four cases, the ${\mathrm{NN}}_{1}^{k}$ spread (first half of iterations) of the distances was larger than the ${\mathrm{NN}}_{2}^{k}$ spread, which indicates the convergence to a minimum. The remaining 12 designs have similar variations of their box plots patterns; compare **S6 Fig**: The smaller ${\mathrm{NN}}_{2}^{k}$ values indicate the focusing on a certain region of sequence space, which differs in all cases from the one chosen by the GA: For 8 of the 16 designs, the mean of the ${\mathrm{NN}}_{2}^{k}$ distances was > 0.4. In summary, we concluded that the GA and the NN protocols find different minima of sequence space in 8 of the 16 designs.

We expected that the NN finds also alternative minima of the FastDesign energy landscape. Thus, we determined for each of the four proteins from *repr_prot* mean $dist_{r}^{\mathrm{FD}}$ values by comparing FD_1000_, i.e., the 1000 sequences generated by FastDesign, with the four sequence sets FD_NN_*r*_ each comprising 250 sequences generated by the NN after training with 250, 500, 750, or 1000 sequences scored by means of FastDesign. The values were added to **Table 2**, are comparable to the differences between the GA and the NN protocols and propose the usage of an NN to broaden the sampling of the FastDesign energy landscape.

### Epistatic effects may cause alternative minima

The region of sequence space optimal for a given protein backbone seems relatively limited to the neighborhood of the native sequence [50]. Although scoring better than native ones, the sequences generated by the GA and the NN for the design shells were markedly different, which prompted us to find an explanation.

One reason could be that the width of sequence sampling differs markedly between the GA and the NN protocol. In order to compare the sampled sequences space, we determined sequence variability of the *s*/2 sequences ($cand_{r}^{\mathrm{GA}},cand_{r}^{\mathrm{NN}}$; see Materials and methods) that were generated by the GA or the NN and used to replace the bottom half of OPT_*r*_ during each iteration *r*. Each sequence from a *cand*_*r*_ set was compared to the most similar one from OPT_*r*_ to determine the number of newly introduced mutations. Sequence variability deduced from 100 iterations is shown in **Table 3** and that of the first 10 iterations in **Table 4**, because the initial iterations are crucial for NN training. Both distributions demonstrate that the GA generated the $cand_{r}^{\mathrm{GA}}$ sequences mainly by introducing single point mutations, whereas the NN introduced up to 18 mutations to generate a $cand_{r}^{\mathrm{NN}}$ sequence. These findings indicate that the NN approach samples sequence space much broader than the GA and generalizes well, because these highly mutated sequences reach high $RS_{3\mathrm{DM}}^{j}$ values. Moreover, these results make clear that the NN is trained with a broad sequence set, because the $RS_{3\mathrm{DM}}^{j}$ values of these novel and diverse $cand_{r}^{\mathrm{NN}}$ sequences are part of the training set utilized by the next iteration *r*+1.

**Table 3:** Comparison of sequence heterogeneity in candidate sequences added to OPT_*r*_ during *r* = 100 iterations.

Mutations	2dri		2ifb		1opb		2rct	
	GA	NN	GA	NN	GA	NN	GA	NN
1	11551	10606	11551	9562	11551	9019	11551	9226
2		708		1527		1738		1456
3		123		259		297		327
4		71		145		98		134
5		38		65		84		99
6		44		37		73		110
7		32		29		99		109
8		31		20		126		62
9		28		19		57		40
10		11		19		28		24
11		14		6		17		31
12		3		7		22		16
13		2		5		10		23
14		4		3		17		26
15		1		1		21		14
16				3		11		12
17				2		11		8
18						3		2

The table lists numbers of candidate sequences $cand_{r=100}^{\mathrm{GA}}$ and $cand_{r=100}^{\mathrm{NN}}$ grouped according to their differences (number of mutations) to the most similar sequence from OPT_*r* = 100_. For this analysis, *r* = 100 iterations of the designs for the four proteins from *repr_prot* were analyzed.

**Table 4:** Comparison of sequence heterogeneity in candidate sequences added to OPT_*r*_ during the first 10 iterations.

Mutations	2dri		2ifb		1opb		2rct	
	GA	NN	GA	NN	GA	NN	GA	NN
1	1067	237	1066	160	1058	77	1059	116
2		442		472		408		373
3		113		149		162		140
4		71		83		72		65
5		38		56		55		44
6		44		37		47		42
7		32		29		42		55
8		31		20		36		41
9		28		19		32		39
10		11		19		28		24
11		14		6		17		31
12		3		7		22		16
13		2		5		10		23
14		4		3		17		26
15		1		1		21		14
16				3		11		12
17				2		11		8
18						3		2

The table lists numbers of candidate sequences $cand_{r=10}^{\mathrm{GA}}$ and $cand_{r=10}^{\mathrm{NN}}$ grouped according to their differences (number of mutations) to the most similar sequence from OPT_*r* = 10_. For this analysis, the first 10 iterations of the designs for the four proteins from *repr_prot* were analyzed.

It is known that mutual dependencies in the occupancy of residue positions drastically affect the fitness landscape as has been confirmed *in silico* [51] and experimentally [52, 53]. As a consequence, high-order epistasis constrains the adaptive pathways that can be followed by evolution [54], because each given occupancy of residue positions severely restrains the subsequently tolerated mutations.

The design shells used here consist of a small number of highly constrained residue positions and the above findings strongly suggest that their occupancy is mutually dependent. Analogously to the evolution of native proteins, each design protocol induces a specific chronological order of residue substitutions and a trajectory that is–under these circumstances–most likely inaccessible, if the order of mutations is different. Thus, if GA and NN choose dissimilar residues for some key positions during the first iterations, the sequence space available to subsequent candidates will be different, if epistasis is dominant. In order to illustrate the existence of epistatic effects, we analyzed mutual dependencies of residue pairs in four design shells.

**Fig 5:**
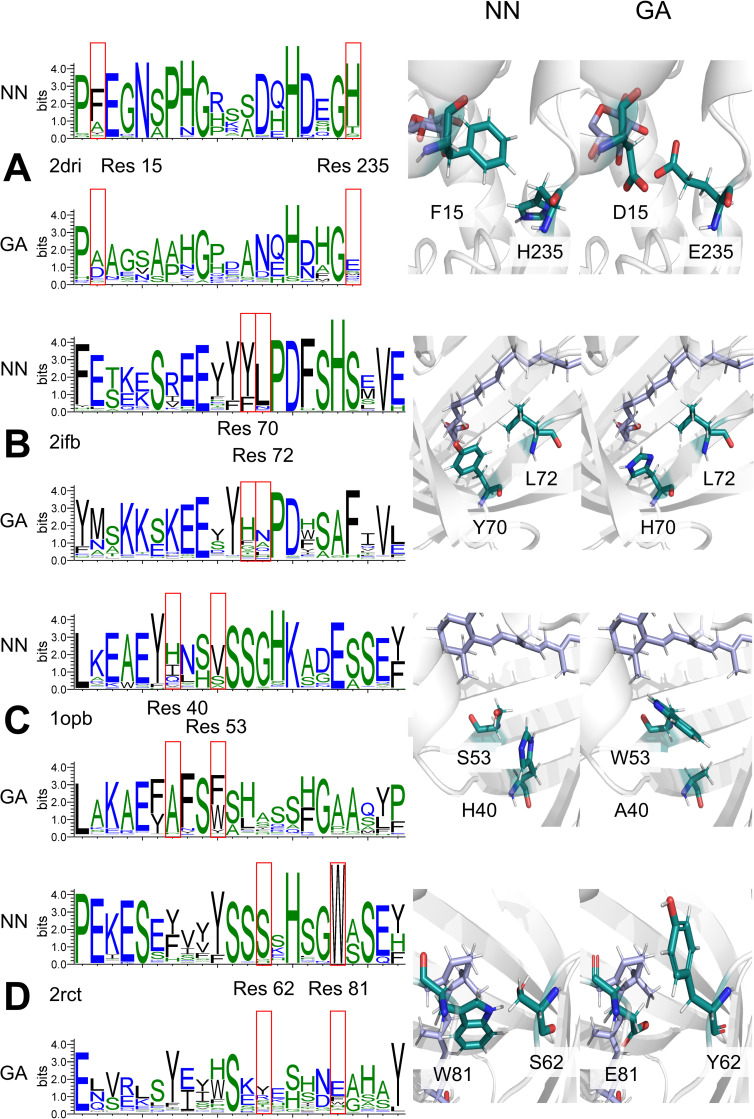
Residue occupancies generated by Rosetta:MSF:GA and Rosetta:MSF:NN for four design shells. For each design shell, the GA- and NN-specific logos resulting from the 239 sequences of generation 100 are given. In each case, one residue pair that is most likely affected by epistatic effects is indicated with red boxes and the 3D orientation of the residues is shown as sticks for the NN (left) and the GA (right) design solution. The designs for the proteins from *repr_prot* were analyzed: (A) 2dri, (B) 2ifb, (C) 1opd, (D) 2rct. The chosen residues are shown in turquoise and the ligand in pale blue; the rest of the protein is represented as a white cartoon.

We manually compared sequence logos resulting from GA and NN generation 100 and searched for positions that were occupied by strikingly different residues. By inspecting the 3D structure of corresponding design candidates, consequences, i.e. pairwise mutual dependencies, of these choices were made plausible. **Fig 5** illustrates differences in the orchestration of the design shells of *repr_prot* proteins generated by GA and NN protocols; all arrangements are valid with respect to orientation and Rosetta scores. In the design shell of 2dri, we found mutual dependencies in the occupancies of positions 15 and 235. The GA chose at position 15 preferentially Ala or Asp and at position 235 Glu. In contrast, the NN preferred at position 15 Phe and at position 235 His. In the design shell of 2ifb, the occupancies of residue positions 70 and 72 are mutually dependent: The GA prefers for position 70 His and for position 72 Asn. The NN chose at position 70 Tyr or Phe and at position 72 Leu. In the design shell of 1opb, the GA selected for position 40 Ala and for position 53 Phe or Trp. In contrast, the NN introduced at position 40 His or Ile and at position 53 Val or Ser. In the design shell of 2rct, the GA chose for position 62 preferentially Tyr and for position 81 Glu. In contrast, the NN preferred at position 62 Ser and at position 81 Trp.

In summary, these analyses illustrate strong mutual dependencies in the occupancies of design shell residues. As design shells are generated by mutating residues in a randomly chosen chronology, epistasis directs the protocols to different regions of sequence space.

### The NN outperforms the GA on multiple mutations and adopts iteratively the energy landscape

A major difference of the NN- and GA-based protocol is the number of mutations introduced per iteration, which are 1 to *n* for the NN and one for the GA approach; compare **Table 3**. Thus, one might argue that the superior performance of the NN-based approach is simply a consequence of the more exhaustive sampling of sequence space resulting from the simultaneously introduced multiple mutations. If the number of mutations has a much stronger effect on performance than the protocol, a modified GA that can introduce up to *n* mutations per iteration, must reach a performance that is comparable to the NN-based protocol. Thus, we created a multi-mutation GA protocol, which we named mmGA, and equipped it with a function that chooses 1 to *n* mutations as does the NN. **Fig 3** makes clear that multi mutations degraded the performance of the GA: Compared to the standard GA that introduces single mutations, the mmGA required twice the number of iterations to find sequences having native-like RS-values.

The training with the tuples generated during the first iteration of the HisB_GA_raw_ design resulted in a relatively low PCC of 0.62 (*p* = 1.2E-9) and the average error was 2.9 REU. This relatively disappointing result argues in favor of utilizing a simpler and much faster regression model that might replace the NN. On the other hand, the results shown in **Fig 5** are indicative of non-linear dependencies in the orchestration of residue positions, which is in agreement with literature [51–53]. In order to test whether the NNs improved their approximation of the energy landscape during the iterative training, we determined for each iteration the PCC of the $RS_{\mathrm{NN}}^{j}$ and the corresponding $RS_{3\mathrm{DM}}^{j}$ values. In **Table 5** the result for the four designs from *repr_prot* are tabulated for the first ten iterations. In all four cases, the initially modest correlation reaches a PCC value of 0.95 not later than after 10 iterations. This finding strongly suggests that the iterative training allowed the NNs to improve their approximation of the energy landscape and argues against a simpler modelling approach.

**Table 5:** Generation-specific PCC values determined for the first ten iterations of four designs.

Generation	2dri	2ifb	1opb	2rct
1	0.53	0.54	0.48	0.42
2	0.84	0.87	0.70	0.69
3	0.90	0.92	0.78	0.74
4	0.95	0.94	0.82	0.82
5	0.97	0.96	0.88	0.89
6	0.98	0.97	0.91	0.92
7	0.98	0.97	0.92	0.94
8	0.98	0.98	0.93	0.94
9	0.98	0.98	0.95	0.96
10	0.98	0.98	0.95	0.96

The table lists Pearson correlation coefficient (PCC) values for the first 10 iterations of the designs with the proteins from *repr_prot* and Rosetta:MSF:NN:enzdes.

### For the NNs, a limited number of samples maps the complexity of a problem-specific energy landscape in sufficient detail

Generally, the reconstruction of an all-atom model from an incomplete representation of a protein is a challenging problem [55]. Thus, at first glance it seems surprising that a one-hot encoded representation of candidate sequences suffices to predict the correct *RS* values with an average error of 1.8 REUs; compare **Fig 2**. To determine an *RS*_3DM_ value, Rosetta has to build a 3D model and to find a combination of rotamers that is suitable for all residues of the design shell. Why is an NN that lacks the assessment of a three-dimensional representation, so successful in an enzdes protocol? Although the NN does not explicitly process three-dimensional information, it can learn the energy landscape, because the *RS*_3DM_ values of the training data implicitly transfer three-dimensional information into the scoring function learned by the NN.

Another example for the beneficial mapping of structure space by means of an NN is refineD, which is aimed at protein structure refinement. This program utilizes additional restraints integrated into a Rosetta all-atom energy function that have been deduced by means of a deep convolutional neural field from the starting structure. refineD outperformed unrestrained relaxation strategies, most likely because these restraints guide conformational sampling [56].

Furthermore, the performance of our NN and our analysis of residue pairs suggests that the orchestration of the design shells with amino acid residues and their orientation is severely biased. This assumption is supported by recent statistical findings related to residue preferences: The algorithm NEPRE assesses successfully the quality of three-dimensional protein models. It is based on a scoring system for residue neighborhood preferences deduced from 14,647 PDB structures. It turned out that certain residues exhibit strong preferences for their neighboring residues and their relative positions [57]. trRosetta generates with high quality the three-dimensional structure of proteins based on predictions for inter-residue contacts, distances, and residue orientations. These predictions originate from a deep residual network that analyzes protein-specific MSAs. For the 31 FM targets of the CASP13 contest, the mean TM-score, which signals the correspondence with the native structure, has been 0.625. The score had dropped only marginally to 0.592, if residue orientation has been ignored during structure prediction [58]. Along this line, a restricted number of residue orientations has been observed at certain positions in more than 2600 antibody structures [59]. Taken together, these findings support the idea that Rosetta:MSF:NN:enzdes performs well, because the orchestration of the design shell with a restricted number of amino acid residues has a stronger effect on scores than the choice of rotamers.

## Supporting information

S1 Fig Grid search varying the number of neurons of the hidden layers. (PDF)

S2 Fig Performance of an NN for two different HisB_GA datasets. (PDF)

S3 Fig Convergence of Rosetta:MSF:GA:enzdes and of Rosetta:MSF:NN:enzdes. (PDF)

S4 Fig Design performance of an alternative residue representation. (PDF)

S5 Fig Design performance with the *talaris* scoring function. (PDF)

S6 Fig Amino acid frequency distributions for the outcome of the first and second half of design protocols. (PDF)

S1 Table One-hot encoding of the 20 amino acid residues. (PDF)

S2 Table Feature vectors of the 20 amino acid residues. (PDF)

S1 Text Structure of the benchmark dataset. (PDF)

S2 Text Additional redesigns to assess the robustness of Rosetta:MSF:NN. (PDF)
