# Supplementary material for: Rosetta:MSF:NN: Boosting performance of multi-state computational protein design with a neural network
Source: PLoS One. 2021 Aug 26;16(8):e0256691. doi: 10.1371/journal.pone.0256691 (PMC8389498; doi:10.1371/journal.pone.0256691)
Supplement: S1 Fig — (PDF) [file pone.0256691.s001.pdf]

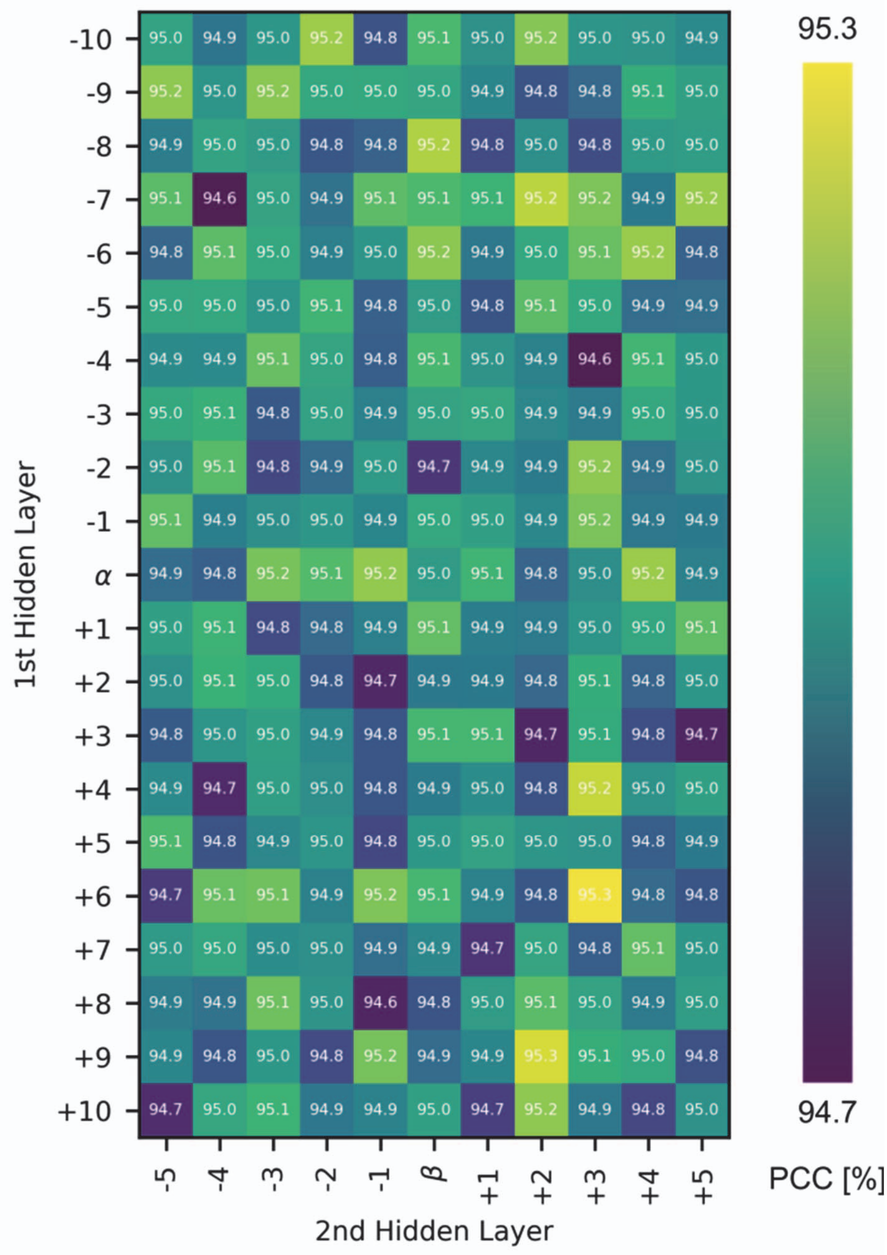

S1 Fig. Grid search varying the number of neurons of the hidden layers. Each cell of this heat map lists the Pearson correlation coefficient (PCC, in percent) resulting from a comparison of the 16,196 pairs of RS_{NN}- and RS_{3DM}-values for the test set sequences from HisB_GA$_{raw}$; see Materials and Methods for a detailed description of the data. For the computation of the RS_{NN}-values, the number of neurons of the two hidden layers was altered systematically based on the number n = 14 of residue positions constituting the design shell. The reference values are $\alpha = 10 + 20n/2$ and $\beta = 5 + 20n/4$. The y-axis denotes the number of neurons used for the first hidden layer and the x-axis the number of neurons used for the second hidden layer. To determine the background colors of the cells, a color scheme violet (lowest value) – yellow (largest value) was used to map the PCC values. The performance did not critically depend on the number of NNs and architectures of the central grid area represented a broad optimum.
