# Supplementary material for: Rosetta:MSF:NN: Boosting performance of multi-state computational protein design with a neural network
Source: PLoS One. 2021 Aug 26;16(8):e0256691. doi: 10.1371/journal.pone.0256691 (PMC8389498; doi:10.1371/journal.pone.0256691)
Supplement: S2 Fig — (PDF) [file pone.0256691.s002.pdf]

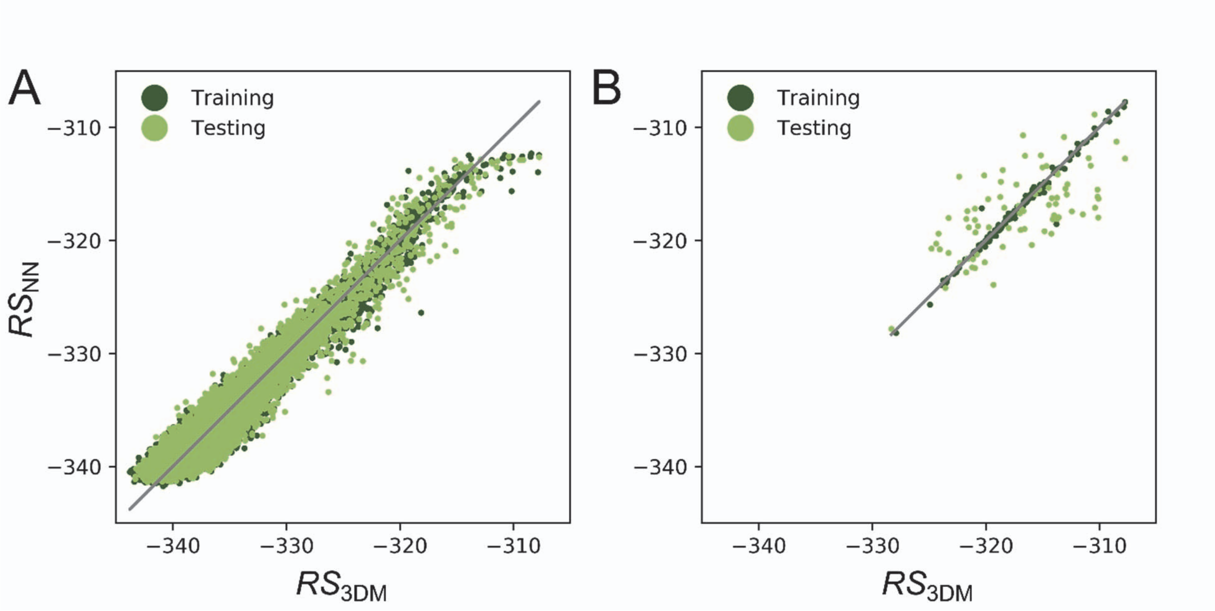

S2 Fig. Performance of an NN for two different HisB_GA datasets. (A) Plot of Rosetta scores RS^{j}_{3DM} deduced from $3D_{opt}$ models versus the Rosetta scores RS^{j}_{NN} predicted by an NN for an enzyme design. Scores are given in REUs and training and test data are represented as dark and light green dots, respectively. The gray line indicates the diagonal, i.e. the position of perfect predictions. The dots represent all of the 32,392 sequences $des\_seq^{raw}_{j}$ of the dataset $\mathrm{HisB\_GA}_{\mathrm{raw}}$; the Pearson correlation coefficient was 0.95. (B) Performance after a training with the 158 $des\_seq^{raw}_{j}$ generated during the first iteration of the GA; the Pearson correlation coefficient was 0.31.
