# Supplementary material for: Rosetta:MSF:NN: Boosting performance of multi-state computational protein design with a neural network
Source: PLoS One. 2021 Aug 26;16(8):e0256691. doi: 10.1371/journal.pone.0256691 (PMC8389498; doi:10.1371/journal.pone.0256691)
Supplement: S3 Fig — (PDF) [file pone.0256691.s003.pdf]

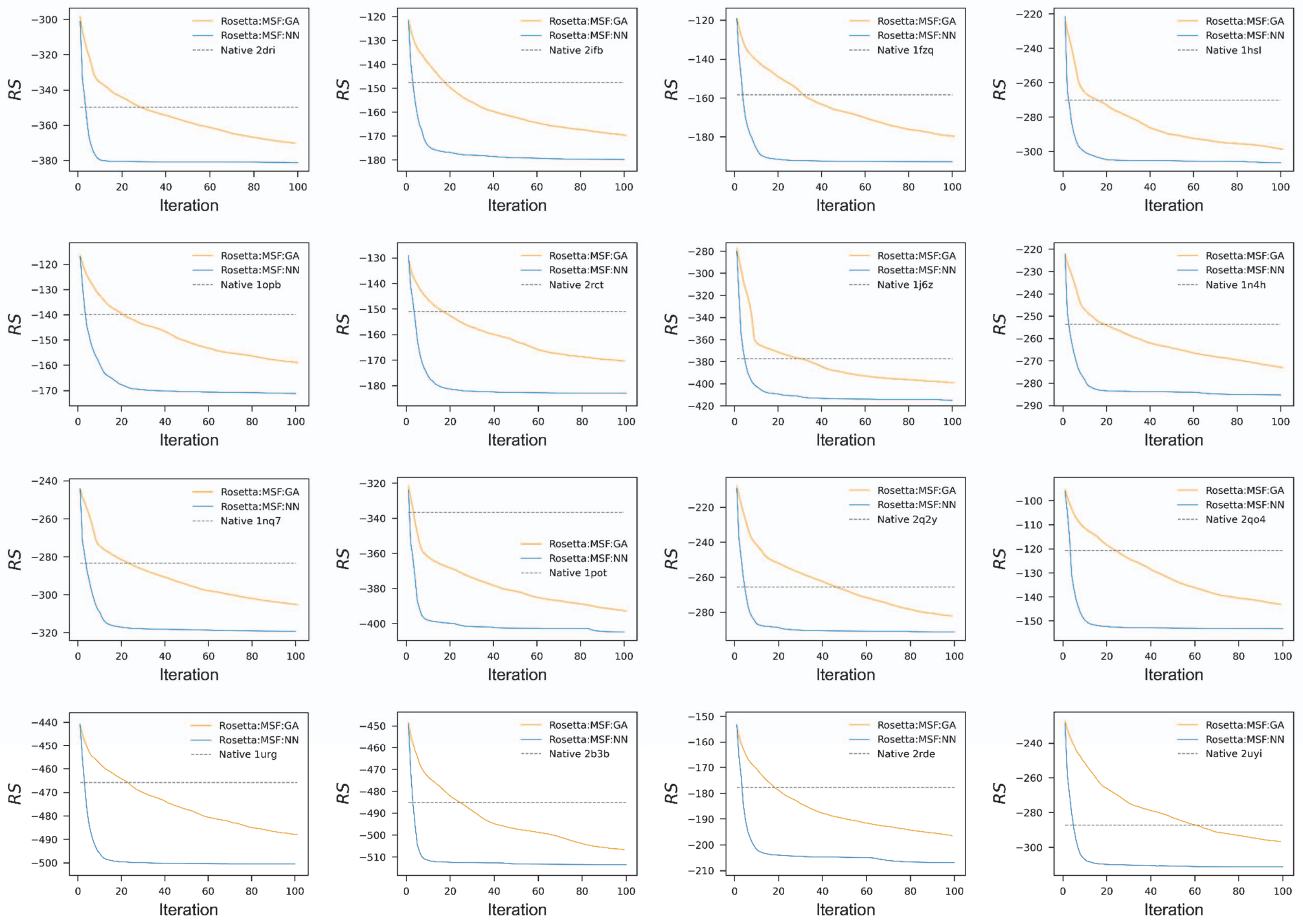

S3 Fig. Convergence of Rosetta:`MSF:GA:enzdes` and of `Rosetta:MSF:NN:enzdes`. For each of the 16 designs, the mean Rosetta scores $RS(\mathrm{OPT}_r)$ were determined in REUs for each iteration r = 1 - 100 and plotted. $RS(\mathrm{OPT}_r)$ is the mean of the Rosetta scores RS^j_{3DM} of all sequences related to the iteration-specific sequence set OPT_r; compare Eq 2. The blue lines represent the $RS_{NN}(\mathrm{OPT}_r)$ and the orange lines the $RS_{GA}(\mathrm{OPT}_r)$ values. The dashed, horizontal line marks the score of the relaxed native protein; the PDB-ID of the corresponding protein is indicated.
