# Supplementary material for: Rosetta:MSF:NN: Boosting performance of multi-state computational protein design with a neural network
Source: PLoS One. 2021 Aug 26;16(8):e0256691. doi: 10.1371/journal.pone.0256691 (PMC8389498; doi:10.1371/journal.pone.0256691)
Supplement: S4 Fig — (PDF) [file pone.0256691.s004.pdf]

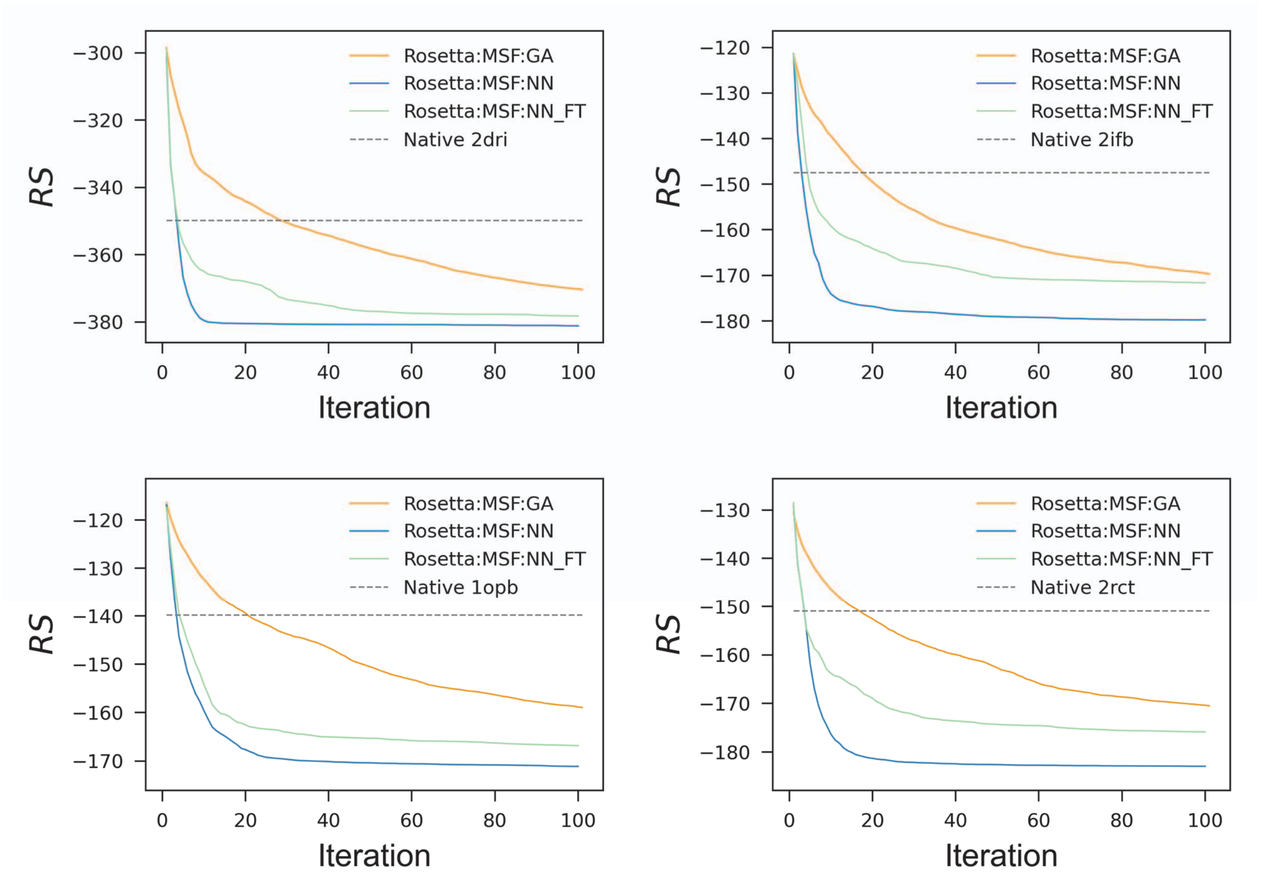

S4 Fig. Design performance of an alternative residue representation. For each of the four proteins from *repr_prot*, **Rosetta:MSF:NN_FT** was used. This protocol utilized for the representation of residues five features listed in S2 Table. The plots of **Rosetta:MSF:GA** and **Rosetta:MSF:NN** served as control and the dotted horizontal line represents the score of the relaxed native protein. All Rosetta scores (RS) are given in Rosetta Energy Units (REU).
