# Supplementary material for: Rosetta:MSF:NN: Boosting performance of multi-state computational protein design with a neural network
Source: PLoS One. 2021 Aug 26;16(8):e0256691. doi: 10.1371/journal.pone.0256691 (PMC8389498; doi:10.1371/journal.pone.0256691)
Supplement: S5 Fig — (PDF) [file pone.0256691.s005.pdf]

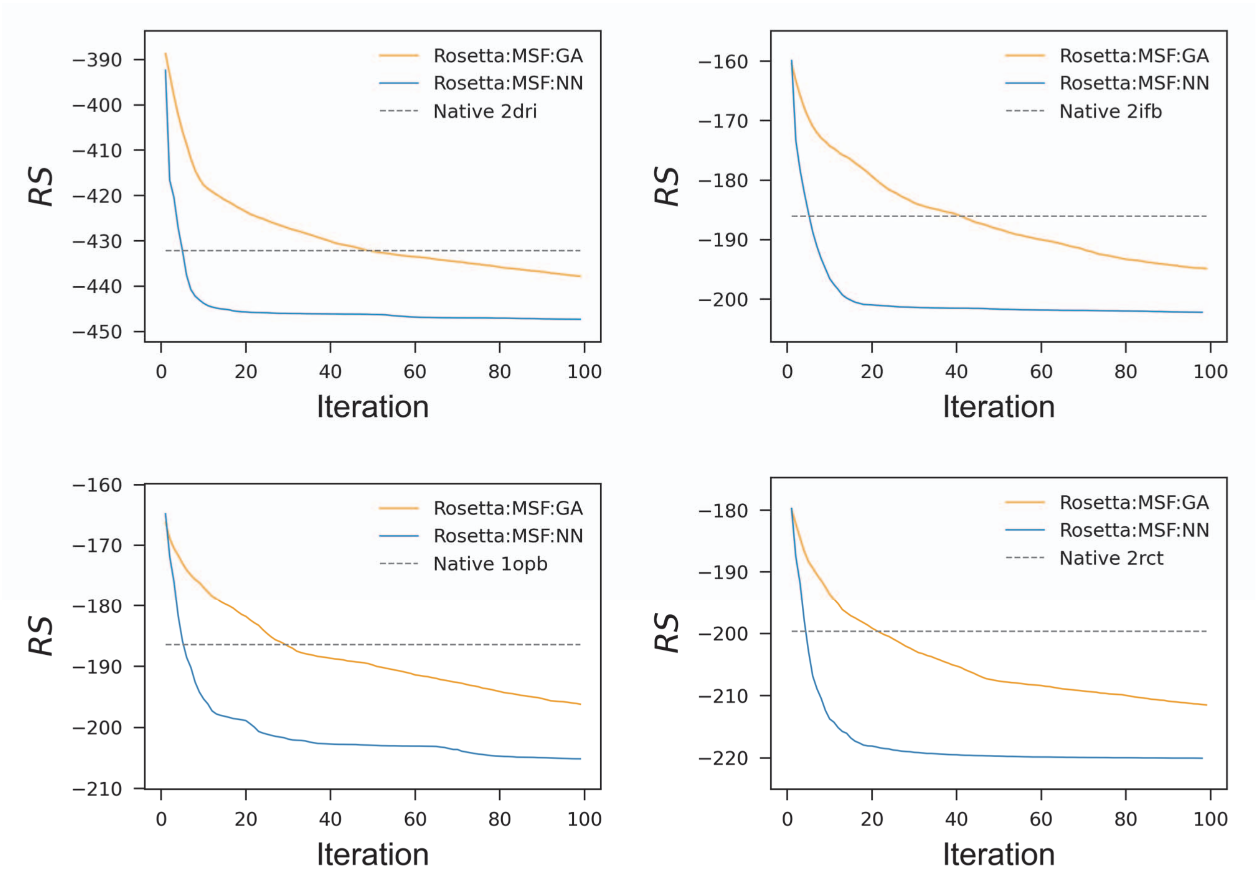

S5 Fig. Design performance with the *talaris* scoring function. For each of the four proteins from *repr_prot*, **`Rosetta:MSF:NN`** was rerun using the scoring function *talaris*. The plots of **`Rosetta:MSF:GA`** served as control and the dotted horizontal line represents the score of the relaxed native protein. All Rosetta scores (RS) are given in Rosetta Energy Units (REU).
