# Supplementary material for: Rosetta:MSF:NN: Boosting performance of multi-state computational protein design with a neural network
Source: PLoS One. 2021 Aug 26;16(8):e0256691. doi: 10.1371/journal.pone.0256691 (PMC8389498; doi:10.1371/journal.pone.0256691)
Supplement: S6 Fig — (PDF) [file pone.0256691.s006.pdf]

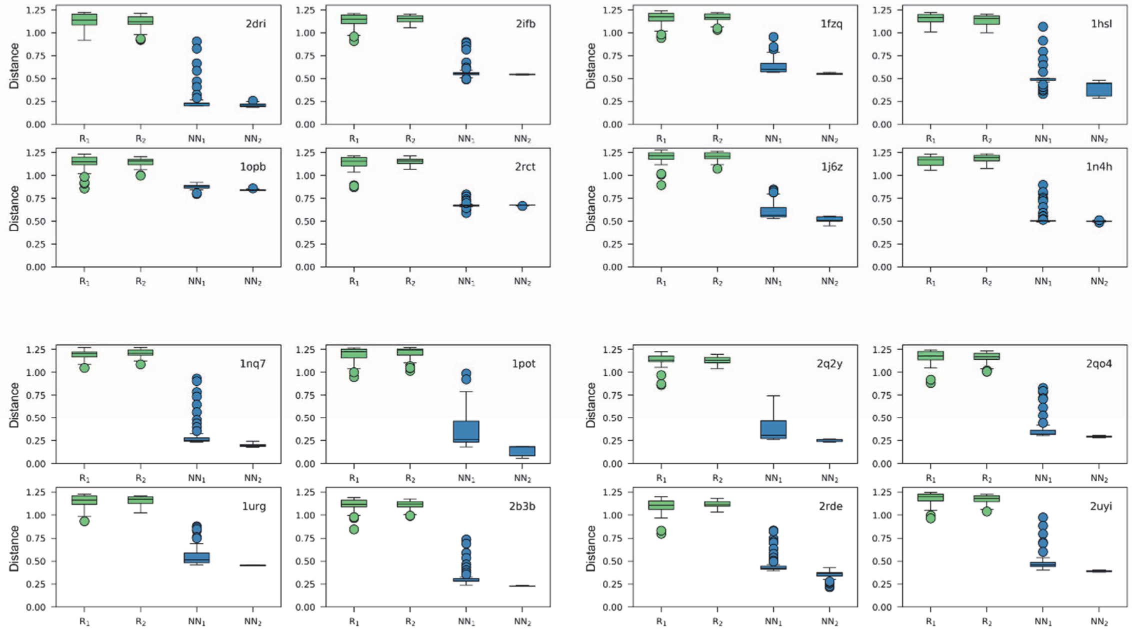

S6 Fig. Amino acid frequency distributions for the outcome of the first and second half of design protocols. The boxplots represent the distributions of Euclidean distances between amino acid frequencies tables $ft_{k,r}$ related to the iterations of the NN based protocol and a reference table ft_k^{GA} from the GA based protocol for the design *k*. The group NN_1^k contains the distributions related to the first 50 and NN_2^k the distributions of the second 50 iterations. For R_1^k and R_2^k the values of each table $ft_{k,r}^{\mathrm{NN}}$ were shuffled; for details see Materials and Methods. The boxplots show the results for the 16 designs of MD_EnzBench.
