# Supplementary material for: Rosetta:MSF:NN: Boosting performance of multi-state computational protein design with a neural network
Source: PLoS One. 2021 Aug 26;16(8):e0256691. doi: 10.1371/journal.pone.0256691 (PMC8389498; doi:10.1371/journal.pone.0256691)
Supplement: S1 Table — (PDF) [file pone.0256691.s007.pdf]

S1 Table. One-hot encoding of the 20 amino acid residues.

Amino acid	Vector representing the amino acid																			
Lys	1	0	0	0	0	0	0	0	0	0	0	0	0	0	0	0	0	0	0	0
His	0	1	0	0	0	0	0	0	0	0	0	0	0	0	0	0	0	0	0	0
Arg	0	0	1	0	0	0	0	0	0	0	0	0	0	0	0	0	0	0	0	0
Asp	0	0	0	1	0	0	0	0	0	0	0	0	0	0	0	0	0	0	0	0
Glu	0	0	0	0	1	0	0	0	0	0	0	0	0	0	0	0	0	0	0	0
Asn	0	0	0	0	0	1	0	0	0	0	0	0	0	0	0	0	0	0	0	0
Gln	0	0	0	0	0	0	1	0	0	0	0	0	0	0	0	0	0	0	0	0
Ser	0	0	0	0	0	0	0	1	0	0	0	0	0	0	0	0	0	0	0	0
Thr	0	0	0	0	0	0	0	0	1	0	0	0	0	0	0	0	0	0	0	0
Cys	0	0	0	0	0	0	0	0	0	1	0	0	0	0	0	0	0	0	0	0
Gly	0	0	0	0	0	0	0	0	0	0	1	0	0	0	0	0	0	0	0	0
Ala	0	0	0	0	0	0	0	0	0	0	0	1	0	0	0	0	0	0	0	0
Pro	0	0	0	0	0	0	0	0	0	0	0	0	1	0	0	0	0	0	0	0
Val	0	0	0	0	0	0	0	0	0	0	0	0	0	1	0	0	0	0	0	0
Met	0	0	0	0	0	0	0	0	0	0	0	0	0	0	1	0	0	0	0	0
Ile	0	0	0	0	0	0	0	0	0	0	0	0	0	0	0	1	0	0	0	0
Leu	0	0	0	0	0	0	0	0	0	0	0	0	0	0	0	0	1	0	0	0
Tyr	0	0	0	0	0	0	0	0	0	0	0	0	0	0	0	0	0	1	0	0
Phe	0	0	0	0	0	0	0	0	0	0	0	0	0	0	0	0	0	0	1	0
Trp	0	0	0	0	0	0	0	0	0	0	0	0	0	0	0	0	0	0	0	1
