# Supplementary material for: Rosetta:MSF:NN: Boosting performance of multi-state computational protein design with a neural network
Source: PLoS One. 2021 Aug 26;16(8):e0256691. doi: 10.1371/journal.pone.0256691 (PMC8389498; doi:10.1371/journal.pone.0256691)
Supplement: S2 Table — (PDF) [file pone.0256691.s008.pdf]

S2 Table. Feature vectors of the 20 amino acid residues.

Amino acid	Volume	Polarity	Isoelectric point	Hydrophobicity	Mean solvent accessibility
**Lys**	68.0	64.2	86.9	43.5	54.3
**His**	49.2	43.2	59.2	23.1	28.1
**Arg**	70.8	51.9	100.0	22.6	50.1
**Asp**	31.3	100.0	0.0	17.5	45.0
**Glu**	47.2	93.8	3.2	17.8	48.6
**Asn**	35.4	63.0	31.3	2.4	46.1
**Gln**	51.3	45.7	34.4	0.0	43.6
**Ser**	18.1	32.1	34.8	1.9	40.5
**Thr**	34.0	21.0	45.7	1.9	35.3
**Cys**	28.0	7.4	26.3	40.3	7.4
**Gly**	0.0	37.0	38.5	2.7	54.0
**Ala**	15.9	25.9	39.2	23.1	37.4
**Pro**	41.0	21.0	40.2	73.5	66.2
**Val**	47.7	8.6	38.5	49.6	19.6
**Met**	62.8	4.9	35.7	44.3	3.9
**Ile**	63.6	0.0	39.2	83.6	7.5
**Leu**	63.6	0.0	38.6	57.6	10.1
**Tyr**	78.5	9.9	34.4	70.8	30.1
**Phe**	77.2	1.2	38.6	76.1	5.5
**Trp**	100.0	4.9	37.7	100.0	13.8

Values are taken from Bogardt RA, Jr., Jones BN, Dwulet FE, Garner WH, Lehman LD, Gurd FR. Evolution of the amino acid substitution in the mammalian myoglobin gene. J Mol Evol. 1980;15(3):197-218. Epub 1980/07/01. doi: 10.1007/bf01732948. PubMed PMID: 7401178.
