# Supplementary material for: Rosetta:MSF:NN: Boosting performance of multi-state computational protein design with a neural network
Source: PLoS One. 2021 Aug 26;16(8):e0256691. doi: 10.1371/journal.pone.0256691 (PMC8389498; doi:10.1371/journal.pone.0256691)
Supplement: S1 Text — (PDF) [file pone.0256691.s009.pdf]

S1 Text. Structure of the benchmark dataset

All required data for the benchmark are available at https://github.com/JulianNazet/Benchmark_Rosetta-MSF-NN. For each protein of the benchmark dataset, ten different PDB files representing the states are included as well as the parameter file of the ligand. Furthermore, it contains all flag files to run the benchmark. As an example, the files related to one design (PDB-ID 1fzq) are listed. The correspondence file (filename 1fzq.corr) maps each mutation to one residue position of the protein. Accordingly, the first resfile (filename 1fzq.resfile) defines which amino acids to use, whereas the second resfile (filename 1fzq.resfile2) describes the repack shell. The fitness function is included in the fitness file (filename fitness.daf). Each state needs to be defined and linked to its PDB file. Finally the default options used for the Rosetta:MSF [1] run are included in the options file (filename MSF_Options).

Additional Reference

1. Löffler P, Schmitz S, Hupfeld E, Sterner R, Merkl R. Rosetta:MSF: a modular framework for multi-state computational protein design. PLoS Comp Biol. 2017;13(6):e1005600. doi: 10.1371/journal.pcbi.1005600.

1fzq.corr:

```
1 23 A
2 25 A
…
20 160 A
```

1fzq.resfile:

```
20
ALLAA EX 1 EX 2
```

1fzq.resfile2:

```
NATRO
START
21 A NATAA EX 1 EX 2
22 A NATAA EX 1 EX 2
…
165 A NATAA EX 1 EX 2
```

fitness.daf:

```
STATE_VECTOR state1 /States/State_1
SCALAR_EXPRESSION best_state1 = vmin( state1 )
STATE_VECTOR state2 /States/State_2
```

```
SCALAR_EXPRESSION best_state2 = vmin( state2 )
…
STATE_VECTOR state10 /States/State_10
SCALAR_EXPRESSION best_state10 = vmin( state10 )
SCALAR_EXPRESSION best_sum = best_state1 + best_state2 + best_state3 + best_state4 + best_state5 + best_state6 + best_state7 + best_state8 + best_state9 + best_state10
FITNESS best_sum
```

MSF_Options:

```
#packing options
-ex1
-ex2
-use_input_sc
-extrachi_cutoff 8
-soft_rep_design
-linmem_ig 42
#minimization options
-run::min_type dfpmin_armijo
-nblist_autoupdate
#score
-score:weights /path/to/rosetta/main/database/scoring/weights/soft_rep_design
#Enzdes
-enzdes::cst_design
-enzdes::design_min_cycles 2
-enzdes::cst_min
-enzdes::lig_packer_weight 1
-enzdes:cst_opt
-enzdes:bb_min
-enzdes:chi_min
-out::nstruct 1
#Params
-extra_res_fa /path/to/specific/params/pdb.params
#MSF
-msf::entity_resfile /path/to/pdb.resfile
-msf::fitness_file /path/to/Fitness.daf
-msf::pop_size YYY
-msf::generations XXX
-msf::fraction_by_recombination 0.05
-msf::seed_sequence_from_input_pdb /path/to/one/state/pdb/.pdb
-msf::resfile_tmpdir /path/to/specific/temp/Tempres
-msf::checkpoint_write_interval 1
-msf::checkpoint_prefix patch/to/Checkpoints/Checkpoint
-msf::seed_sequence_using_correspondence_file path/to/pdb.corr
#-msf::darwin_resume true
```
