# Supplementary material for: Rosetta:MSF:NN: Boosting performance of multi-state computational protein design with a neural network
Source: PLoS One. 2021 Aug 26;16(8):e0256691. doi: 10.1371/journal.pone.0256691 (PMC8389498; doi:10.1371/journal.pone.0256691)
Supplement: S2 Text — (PDF) [file pone.0256691.s010.pdf]

S2 Text. Additional redesigns to assess the robustness of `Rosetta:MSF:NN`.

The specific representation of amino acid properties has little effect on the performance of `Rosetta:MSF:NN`

By applying one-hot encoding (compare S1 Table), each residue occupancy is represented by a 20-column vector having a “1” at the chosen residue and 19 “0” for the other ones. We wanted to make plausible that the performance of `Rosetta:MSF:NN` does not critically depend on this specific residue representation and is robust to limitations of feature tables. Thus, we examined the performance of our program during an alternative recapitulation experiment of the four proteins (set *repr_prot*) with PDB-IDs 2dri, 2ifb, 1opb, and 2rct. During the experiment named `NN_FT`, an alternative representation of residues by means of five features was used. These properties were “volume”, “polarity”, “isoelectric point”, “hydrophobicity”, and “mean solvent accessibility” [1]. S2 Table lists the encoding of the amino acids. S4 Fig shows for each of the four proteins the plot of the Rosetta scores during 100 iterations and in comparison to `Rosetta:MSF:GA` and `Rosetta:MSF:NN`, which served as references. The analysis of the plots resulting from the `Rosetta:MSF:NN_FT` protocol indicates that the specific representation of residues has no drastic effect on the performance of the NN. In all cases, the NN-based algorithm converges faster than the GA-based one, with one-hot encoding reaching the lowest score levels. Note that the user can easily replace the feature table in accordance with his requirements.

`Rosetta:MSF:NN` performs well regardless of the scoring function

We wanted to make plausible that the performance boost of `Rosetta:MSF:NN` does not depend on the chosen scoring function. Therefore, we replaced the *soft-rep* design function which we utilized for all design and benchmark runs, with the *talaris* scoring function [2]. Again, recapitulation experiments were performed for the four proteins from *repr_prot*. S5 Fig shows for each of the four proteins the plot of the Rosetta scores during 100 iterations of `Rosetta:MSF:GA` and `Rosetta:MSF:NN`. The analysis of the plots indicates that in all cases the NN approach outperforms the GA also with the *talaris* scoring function.

Additional References

1. Yousef A, Charkari NM. A novel method based on physicochemical properties of amino acids and one class classification algorithm for disease gene identification. J Biomed Inform. 2015;56:300-306. Epub 2015/07/07. doi: 10.1016/j.jbi.2015.06.018.
2. Leaver-Fay A, O'Meara MJ, Tyka M, Jacak R, Song Y, Kellogg EH, et al. Scientific benchmarks for guiding macromolecular energy function improvement. Methods Enzymol. 2013;523:109-143. doi: 10.1016/B978-0-12-394292-0.00006-0.
